# Characterization of spelt wheat (*Triticum spelta* L.) genotypes using DArTseq technology

**DOI:** 10.1007/s13353-025-01037-4

**Published:** 2025-12-27

**Authors:** Aleksandra Pietrusińska-Radzio, Anna Bilska-Kos, Jan Bocianowski

**Affiliations:** 1 Plant Breeding and Acclimatization Institute—National Research Institute, Radzików, Błonie, 05-870 Poland; 2 Department of Mathematical and Statistical Methods, Poznań University of Life Sciences, Wojska Polskiego 28, Poznań, 60-637 Poland

**Keywords:** Association analysis, DArTseq, Genetic mapping, Genetic similarity, Spelt wheat

## Abstract

The aim of this study was to apply DArTseq technology to analyze *T. spelta* L. (spelt wheat) genotypes in order to eliminate duplicates in the gene bank and ensure the high quality and purity of the stored material. The research included the analysis of genetic similarity, the construction of dendrograms, and association mapping, which enabled the identification of specific molecular diagnostic markers for spelt wheat. Spelt is an ancient cereal species gaining popularity, especially in organic farming. It is characterized by natural resistance to biotic factors and tolerance to environmental stress. Spelt is a valuable material in plant resistance breeding aimed at developing varieties resistant to diseases and well adapted to unfavourable environmental conditions. In this study, molecular characterization of 27 spelt genotypes was carried out using high-throughput DArTseq technology, enabling simultaneous analysis of SilicoDArT and SNP markers. A total of 96,136 markers were identified, of which 16,712 met the quality criteria and were used for genetic similarity and association mapping. Based on similarity coefficients, a dendrogram was created, distinguishing four main genotype groups. Association mapping revealed over 2,600 markers significantly associated with the virulence level of the *B. graminis* f. sp. *tritici* pathogen. Particular attention was paid to SilicoDArT 7,492,586 and SNP 1,126,088 markers, showing significant associations with plant response to three of the five analyzed isolates. Chromosomal regions (1D, 3D, 5B, 6 A) associated with resistance were also identified, confirming the polygenic nature of this trait. Results indicate high genetic variability of the analyzed material and the usefulness of DArTseq technology in identifying markers for resistance breeding. The presented markers can be used in marker-assisted breeding programs, especially considering the growing interest in spelt as a cereal for organic farming. These findings provide a valuable basis for further improvement of spelt resistance and sustainable cereal breeding.

**Supplementary Information:**

The online version contains supplementary material available at 10.1007/s13353-025-01037-4.

## Introduction

Spelt (*Triticum spelta* L.) is an ancient cereal species that is currently gaining popularity, especially in organic programs. In comparison to ancient cereal species, spelt is distinguished by its high nutritional content characterized by increased content of fiber and protein (Kohajdova and Karovicova 2008; Lacko-Bartošová et al. 2010). In resistance breeding, *T. spelta* is used as an effective source of resistance to biotic factors and tolerance to abiotic stress. All these factors influence the fact that spelt is an alternative to organic farming, focused on breeding plants resistant to fungal diseases and able to adapt to unfavorable environmental conditions (Bonafaccia et al. 2000). To use the potential of spelt in breeding, it is the detailed characterization of genetic diversity that is crucial for the ability of spelt to adapt to stressful conditions. Acclimatization of spelt to the inducing factors is essential for the long-term survival of spelt (Dreisigacker et al. 2004; Voss-Fels et al. 2015). Molecular characterization of genotypes enables a detailed identification of genetic profiles that can be utilized in breeding programs (Sheikh et al. 2014; Khan et al. 2015).

Powdery mildew of cereals and grasses, caused by *Blumeria graminis* f. sp. *tritici*, is one of the most serious fungal diseases affecting cereals, including common wheat and spelt (Savary et al. 2019). The disease causes severe leaf damage, leading to yield losses of up to 20–40%, especially when infection involves the flag leaf and ear at early developmental stages, such as the onset of heading (Conner et al. 2003). In cases of severe and early infection, the main cause of losses is a reduction in thousand grain weight. Sources of resistance to powdery mildew of cereals and grasses in spelt include various genes and alleles. The resistance allele *Pm* 1 d was detected in *T. spelta* var. *duhamelianum* (Hsam et al. 1998), while a recessive resistance gene was found on chromosome 2D in the Swiss cultivar ‘Hubel’ (Peng et al. 2014). Additionally, a QTL associated with adult plant resistance was detected on chromosome 5 A of the cultivar ‘Oberkulmer’ (Keller et al. 1999). Pathogenesis and infection-promoting conditions: *B. graminis* f. sp. *tritici* infects leaves, limiting photosynthesis and weakening plants. Infection is favored by high relative humidity (50–100%), moderate temperatures, and short periods of leaf wetness caused by dew or fog. Spring infections lead to weakened winter wheat and reduced yields (Simeone et al. 2020), while mild and wet winters favor the spread of the pathogen (Juroszek and Tiedemann 2013). Wheat resistance to *B. graminis* f. sp. *tritici* is based on the classic “gene-for-gene” concept (Flor 1956), according to which specific plant resistance genes correspond to specific virulence genes of the pathogen. No wheat variety exhibits resistance to all races of the pathogen, and no race infects all wheat genotypes (Czembor 1981; Gacek and Czembor 1983). The importance of monitoring and pyramiding resistance genes: Monitoring *B. graminis* f. sp. *tritici* populations is crucial for identifying effective resistance genes (*Pm* – Powdery mildew) that can be introduced into new varieties. Pyramiding *Pm* genes increases the durability of powdery mildew of cereals and grasses resistance, although gene interactions and pathogen evolution may limit its long-term effectiveness (Pilet-Nayel et al. 2017; Dormatey et al. 2020).

In order to maintain high quality control and prevent the formation of hybrid species, effective molecular tools are needed that could be used by breeders to distinguish spelt wheat from other cultivated wheat species. Preliminary studies indicate that available molecular markers for this species have low diagnostic power and, in many cases, do not correlate with the phenotype. Moreover, many spelt varieties have undergone genetic erosion due to the lack of proper preservation of plant material, protecting it from extinction or contamination with other wheat species (Wiwart and Perkowski 2005; Čurná and Lacko-Bartosova 2017).

The intensive development of molecular biology, combined with resistance breeding, offers new opportunities. The rapid progress in molecular biology techniques enables genotyping using a range of classical markers as well as modern methods based on sequencing. The DNA analysis platform – Diversity Arrays Technology Pty. Ltd. – offers analyses based on next-generation sequencing (NGS) technology – DArTseq (von Cruz et al. 2013; Piechota et al. 2017). The advantage of using this methodology is that 90% of the DArTseq markers are complementary to unique genome sequences (Courtois et al. 2013; von Cruz et al. 2013). DArTseq analysis generates two datasets: the first contains dominant markers, and the second includes codominant markers with detailed single nucleotide polymorphisms. The result is at least three times as many dominant markers as those obtained with conventional DArT methods (Sansaloni et al. 2011; Piechota et al. 2017). The use of high-throughput technologies has enabled the rapid and reliable identification of plant material for important and diverse traits. Furthermore, next-generation sequencing significantly reduces limitations, opening up new research and application opportunities (Tanksley and McCouch 1997; McCouch et al. 2012; Piechota et al. 2017). The DArTseq technology has been used in many crop species for genetic diversity analysis (Baloch et al. 2017), genome-wide association studies (GWAS) (Singh et al. 2018; Visioni et al. 2018), and QTL mapping (Haghdoust et al. 2018).

Over the past decade, the DArT platform has generated two types of markers: SilicoDArT and DArTSeq SNP. SilicoDArT markers are dominant and scored based on the presence or absence of a single allele, while DArTSeq SNP markers are codominant and are generated by detecting DNA polymorphisms and SNPs in reduced genome fragments after enzymatic digestion and NGS sequencing. Both types of markers have been successfully used in various crop species for genetic diversity studies (Yang et al. 2006; Bolibok-Brągoszewska et al. 2009; Sánchez-Sevilla et al. 2015; Tang et al. 2015), genetic mapping (Mace et al. 2008; Schouten et al. 2012; Alam et al. 2014; Ambawat et al. 2016), and population structure analysis (Matthies et al. 2012; Laidò et al. 2013). SNP markers are scored as reference homozygote, alternative homozygote, or heterozygote, which increases their usefulness in genetic population analyses (Kilian et al. 2012; von Cruz et al. 2013).

Thanks to the high density and complementarity of markers, the DArT platform enables accurate genome coverage, which is crucial for effective genomic research, genetic diversity analysis, and conservation and breeding activities in agriculture. The codominant inheritance pattern of SNP markers further expands the analytical capabilities of the platform, enabling detailed population studies and genotype-phenotype linkage analysis (Kilian et al. 2003, 2012; Xia et al. 2005; Dierig et al. 2009).

Association mapping and selective genomics are most commonly used to identify markers and the genes linked to them (Meuwissen et al. 2001; Rakoczy-Trojanowska et al. 2017a, b; Rio et al. 2019; Bocianowski et al. 2023). Association mapping includes two approaches: candidate gene association and genome-wide association studies (GWAS). In the case of candidate gene association, we examine the relationship between DNA polymorphisms in a selected gene and a specific trait. When detailed biochemical knowledge about the trait is lacking, a GWAS analysis is performed. This approach allows for the search of trait-marker associations across the entire genome. During GWAS analysis, it is assumed that markers responsible for the expression of a given trait, which is characterized by linkage disequilibrium (LD), are present in the genome (Bocianowski et al. 2023). In association mapping, this means that certain markers are linked to the studied trait because they are located near genes on the chromosome. They are inherited together regardless of their position on the chromosome, which significantly facilitates the identification of markers associated with traits (Huang et al. 2021; Jain et al. 2024).

The aim of this study is to apply DArTseq technology to analyze *T. spelta* L. (spelt wheat) genotypes in order to eliminate duplicates in the gene bank and ensure the high quality and purity of the stored material. The research includes the analysis of genetic similarity, the construction of dendrograms, and association mapping, which will enable the identification of specific molecular diagnostic markers for spelt wheat. The obtained results will contribute to a better understanding of the genetic diversity of this species and the potential utilization of selected genotypes in breeding and biotechnological programs.

## Materials and methods

### Plant material

The study utilized 27 genotypes of spelt wheat, including 21 genotypes from National Centre for Plant Genetic Resources, Plant Breeding and Acclimatization Institute – National Research Institute (KCRZG IHAR-PIB) and six genotypes obtained from the Leibniz Institute of Plant Genetics and Crop Plant Research (IPK Genebank). Detailed information on the origin of the plant material is provided in Table 1.

In addition, three control genotypes were included in the experiments. In the infection assays, the common wheat (*Triticum aestivum* L.) cultivar ‘Nimbus’ was used in two variants: infected with *B. graminis* f. sp. *tritici* (Bgt) and uninfected (negative control). For association mapping, two reference genotypes were used: the Polish spelt wheat cultivar ‘Rokosz’ (*T. aestivum* ssp. *spelta*), provided by Hodowla Roślin Strzelce Sp. z o.o., and the common wheat cultivar ‘Chinese Spring’ (*T. aestivum* L.) obtained from Diversity Arrays Technology Pty Ltd (Diversity Arrays Technology n.d.).

**Table 1:** Characteristics and sources of spelt wheat accessions used for analysis

No	Accession number	Accession name	Storage source	Country of orgin	Scientific name	Doi
1	1150	Weisser W.Grann. aus Hohenheim	KCRZG IHAR-PIB	not available (n/a)	*Triticum spelta* L. subsp. *spelta var. arduini* Körn.	not available (n/a)
2	1151_1	Weißer Kolben (Wagger, Hohenheim)	KCRZG IHAR-PIB	(n/a)	*Triticum spelta* L. subsp. *spelta var. album* (Alef.) Körn.	(n/a)
3	1151_2	Weißer Kolben (Wagger, Hohenheim)	KCRZG IHAR-PIB	(n/a)	*Triticum spelta* L. subsp. spelta var. *album* (Alef.) Körn.	(n/a)
4	1157	Spelt ‘InZ. Droogendijk/39’	KCRZG IHAR-PIB	(n/a)	not available (n/a)	(n/a)
5	1161	Rottweiler Frühkorn	KCRZG IHAR-PIB	(n/a)	*Triticum spelta* L. subsp. *spelta var. duhamelianum* (Mazzuc.) Körn.	(n/a)
6	1162	Rottweiler Dinkel ST.6	KCRZG IHAR-PIB	(n/a)	*Triticum spelta* L. subsp. *spelta var. duhamelianum* (Mazzuc.) Körn.	(n/a)
7	1165	Red Winter	KCRZG IHAR-PIB	USA	*Triticum spelta* L. subsp. spelta var. duhamelianum (Mazzuc.) Körn.	(n/a)
8	1171	Farnsburg 6	KCRZG IHAR-PIB	(n/a)	*Triticum spelta* L. subsp. *spelta var. duhamelianum* (Mazzuc.) Körn.	(n/a)
9	1172	Burgdorf 1	KCRZG IHAR-PIB	(n/a)	*Triticum spelta* L. subsp. *spelta var. album* (Alef.) Körn.	(n/a)
10	1173	Bregenzer Roter Spelz	KCRZG IHAR-PIB	(n/a)	*Triticum spelta* L. subsp. *spelta var. duhamelianum* (Mazzuc.) Körn.	(n/a)
11	1174	Brauner W. Gran. aus Nördlingen	KCRZG IHAR-PIB	(n/a)	*Triticum spelta* L. subsp. *spelta var. vulpinum* (Alef.) Körn.	(n/a)
12	1175	Brauner Spelt aus Schefflenz	KCRZG IHAR-PIB	(n/a)	*Triticum spelta* L. subsp. *spelta var. duhamelianum* (Mazzuc.) Körn.	(n/a)
13	1179	Kipperhaus Weißer Spelz	KCRZG IHAR-PIB	(n/a)	*Triticum spelta* L. subsp. *spelta var. album* (Alef.) Körn.	(n/a)
14	2638	no data	KCRZG IHAR-PIB	(n/a)	*Triticum spelta* L. subsp. *spelta var. album* (Alef.) Körn.	(n/a)
15	4597	Bauländer Spelz	KCRZG IHAR-PIB	(n/a)	*Triticum spelta* L. subsp. *spelta var. duhamelianum* (Mazzuc.) Körn.	(n/a)
16	4708	Zeiners Weißer Schlegeldinkel	KCRZG IHAR-PIB	(n/a)	*Triticum spelta* L. subsp. *spelta var. album* (Alef.) Körn.	(n/a)
17	4742	Oberländer Spelz	KCRZG IHAR-PIB	(n/a)	*Triticum spelta* L. subsp. *spelta var. duhamelianum* (Mazzuc.) Körn.	(n/a)
18	21,805	Spelz aus Tsari Brod; Brod T. Spelta.	KCRZG IHAR-PIB	(n/a)	*Triticum spelta* L. subsp. *spelta var. album* (Alef.) Körn.	(n/a)
19	21,979	4	KCRZG IHAR-PIB	(n/a)	(n/a)	(n/a)
20	237,611	no data	KCRZG IHAR-PIB	(n/a)	*Triticum spelta* L. subsp. *spelta var. vulpinum* (Alef.) Körn.	(n/a)
21	508,089	no data	KCRZG IHAR-PIB	Nepal	*Triticum spelta* L. subsp. *spelta var. alefeldii* Körn.	(n/a)
22	TRI 251	Müllers Gaiberger	IPK	Germany (before 1945)	*Triticum spelta* L. subsp. *spelta var. duhamelianum* (Mazzuc.) Körn.	10.25642/IPK/GBIS/251
23	TRI 27,769	Spelz, Dinkel	IPK	Iran, Islamic Republic	*Triticum spelta* L. subsp. *spelta var. vulpinum* (Alef.) Körn.	10.25642/IPK/GBIS/258477
24	TRI 27,796	COLL. BBA 1305	IPK	Spain	*Triticum spelta* L. subsp. *spelta var. rubrivelutinum* Körn.	10.25642/IPK/GBIS/244717
25	TRI 27,893	Spelz, Dinkel	IPK	Iran, Islamic Republic	*Triticum spelta* L. subsp. *spelta var. caeruleum* (Alef.) Körn.	10.25642/IPK/GBIS/243018
26	TRI 304	Fuggers Babenhauser Zuchtveesen	IPK	Germany (before 1945)	*Triticum spelta* L. subsp. *spelta var. duhamelianum* (Mazzuc.) Körn.	10.25642/IPK/GBIS/304
27	TRI 9681 (W 1591)	no data	IPK	Morocco	*Triticum spelta* L. subsp. *spelta var. album* (Alef.) Körn.	10.25642/IPK/GBIS/9653

### Evaluation of spelt wheat genotype resistance and maintenance of ***Blumeria graminis*** f. sp. ***tritici*** isolates

To verify virulence and infection intensity, isolates were periodically tested using the Synergy H1 reader (BioTek, Agilent, USA), ensuring stable pathogenic activity throughout the experiments.

#### Assessment of spelt wheat response to infection

**Fig. 1:**
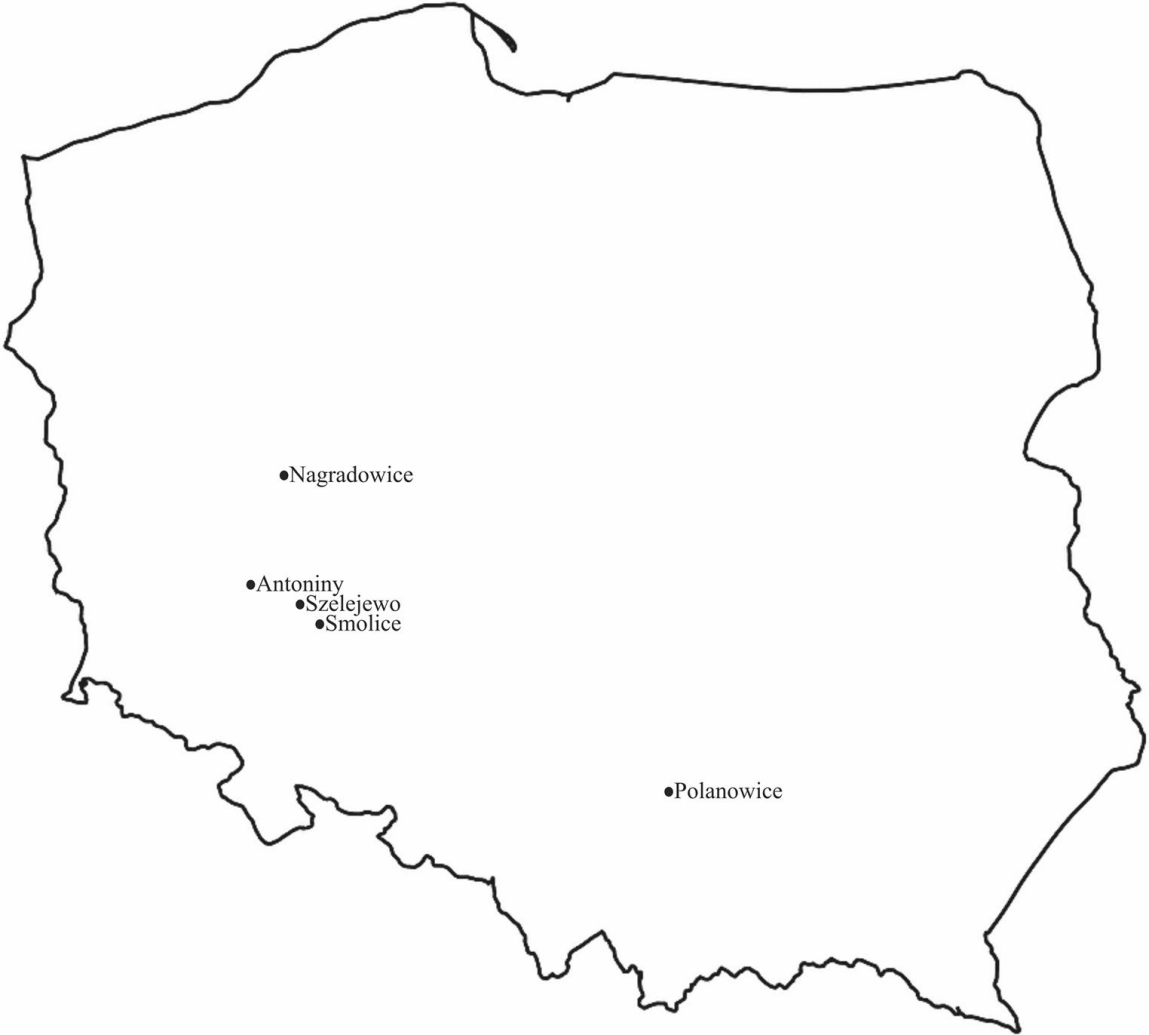
Geographic locations in southwestern Poland where wheat leaves infected by *B. graminis* f. sp. *tritici* were collected in 2017. Sampling sites: Szelejewo, Polanowice, Nagradowice, Antoniny, and Smolice

Resistance of spelt wheat (*T. aestivum* ssp. *spelta*) genotypes to powdery mildew of cereals and grasses was evaluated using five differentiating isolates of *B. graminis* f. sp. *tritici* (Bgt). The isolates originated from infected wheat leaves collected in southwestern Poland in 2017. In 2017, leaf samples infected by the pathogen *B. graminis* f. sp. *tritici* were collected in the following locations: Szelejewo, Polanowice, Nagradowice, Antoniny, and Smolice. The isolates are maintained in the reference collection of the National Centre for Plant Genetic Resources in Radzików (Poland). To preserve their viability and virulence, they are renewed monthly and stored under controlled conditions in a phytotron at 4 °C with a 16-hour light cycle. Each isolate is routinely passaged on the susceptible common wheat cultivar ‘Nimbus’. A figure (Fig. 1) illustrating the geographical distribution of these sampling sites has been included in the manuscript.

Infection assays were carried out at the fully expanded second leaf stage (BBCH 12), ten days after sowing. Each of the five isolates was applied separately by transferring conidia from infected ‘Nimbus’ plants onto the leaves of the tested spelt genotypes. Inoculations were performed under controlled phytotron conditions: plants were kept in darkness for 12 hours at 18 °C with increased humidity, followed by cultivation under a 16-hour light and 8-hour dark cycle at 16–22°C. The first disease symptoms appeared approximately 8–10 days after inoculation.

Phenotypic responses were assessed using the five-point Mains and Dietz (1930) scale, which describes the progression of powdery mildew infection from the absence of visible symptoms, through slight and expanding necrosis with limited sporulation, to the development of chlorosis and dense mycelium with intensive sporulation, typical of fully susceptible reactions.

Genotypes showing scores of 0–2 were considered resistant, whereas those with scores of 3–4 were classified as susceptible. Resistance tests were performed in six replicates for each Bgt isolate. The cultivar ‘Nimbus’ served as both a positive (infected) and negative (uninfected) control to ensure inoculation uniformity and to provide a reliable reference for disease response assessment.

The resulting phenotypic data formed the basis for subsequent statistical analyses of spelt wheat resistance to powdery mildew cereals and grasses.

#### DNA extraction and DArTseq platform analysis

For the molecular studies, genomic DNA was isolated from 27 samples in two replicates. Genomic DNA was extracted using a protocol based on the DNeasy Plant Mini Kit (QIAGEN), with modifications. Approximately 200–250 mg of plant tissue was homogenized using a ball mill (model MM301, Retsch, Verder, Germany) and shaken at a frequency of 30 Hz in four five-minute cycles. After grinding, the entire sample was thoroughly agglomerated and incubated in a TS thermomixer (BioSan, Thermo-Shaker SC-24 C, Latvia) with vigorous shaking on a heating block at 60 °C for 35–40 min. Following this step, the procedure adhered to the DNeasy Plant Mini Kit (QIAGEN) protocol.

Using a NanoDrop 1000 system (Thermo Scientific), absorbance measurements were used to automatically calculate DNA concentration from a ≤ 1.5 µl sample droplet. The quality and quantity of genomic DNA were also assessed by electrophoresis on a 0.7% agarose gel containing ethidium bromide (Agarose NEEO ultra-quality, ROTH) at 200 V in 0.5 × TBE buffer. Electrophoresis images were captured using a UV transilluminator and the Kodak Gel Logic 200 Imaging System (Eastman Kodak Company, Rochester, NY, USA), and image analysis was performed with Kodak Molecular Imaging Software, Version 4.0 (Eastman Kodak Company, Rochester, NY, USA). All DNA samples were subsequently diluted to a final concentration of 50 ng/µl. The genomic DNA isolated using this method was used as the template for next-generation sequencing based on the DArTseq platform. Genome complexity reduction involved digestion with restriction enzymes followed by sequencing of short reads. Molecular analyses were conducted at Diversity Arrays Technology, University of Canberra, Australia. The methods used are described in detail on the Diversity Arrays Technology website: https://www.diversityarrays.com/technology-and-resources/dartseq/, accessed on February 4, 2025. The sequencing results enabled the generation of two types of molecular markers: SilicoDArTs (dominant markers) and SNPs (co-dominant markers, including single and double nucleotide polymorphisms).

#### Statistical analysis

Phenotypic similarity among 27 spelt wheat genotypes was estimated based on Euclidian similarity of resistance profiles and grouped using the unweighted pair group method with arithmetic mean (UPGMA) method. Genetic similarity among 27 spelt wheat genotypes was estimated based on molecular marker observations. The calculated Nei similarity coefficients (Nei 1972) were used to group the genotypes using the unweighted pair group method with arithmetic mean (UPGMA) method. The results of the groupings were presented as dendrogram. Additionally, principal component analysis (PCA) was used to present the genetic relationship between plant lines in the spelt collection. LD measurements were conducted on the basis of SilicoDArT markers demonstrating significant linkage disequilibrium (LD), defined by a *p*-value threshold of less than 0.01. The assessment of LD decay involved plotting pairwise LD values (*r*^2^) against the physical distance between SNPs. This analysis was performed for segments of 300 kilobases (kb) within individual chromosomes and across the entire genome, utilizing nonlinear regression. Based on the results obtained from genotyping and phenotyping, association mapping was performed using GWAS analysis. For the association analysis, only SilicoDArT and SNP sequences meeting the following criteria were selected: one SilicoDArT or SNP (or both) within a given sequence (69 nt), minor allele frequency (MAF) > 0.25, and the missing observation fractions < 10%. Association mapping, based on SilicoDArT and SNP markers and average level of virulence of *B*. *graminis* f. sp. *tritici*, was performed separately for five isolates using a method based on a mixed linear model with a population structure estimated by eigen analysis and modeled by random effects (van Eeuwijk et al. 2010; Malosetti et al. 2013; Bocianowski and Leśniewska-Bocianowska 2025). All analyses and visualizations of the results were performed in GenStat 23.1 (VSN International 2023), using the QSASSOCIATION procedure (Patterson et al. 2006). The significance of the association between level of virulence of *B*. *graminis* f. sp. *tritici* and molecular markers was assessed using *p*-values corrected for multiple testing using the Benjamini–Hochberg method (Benjamini and Hochberg 1995).

## Results

**Fig. 2:**
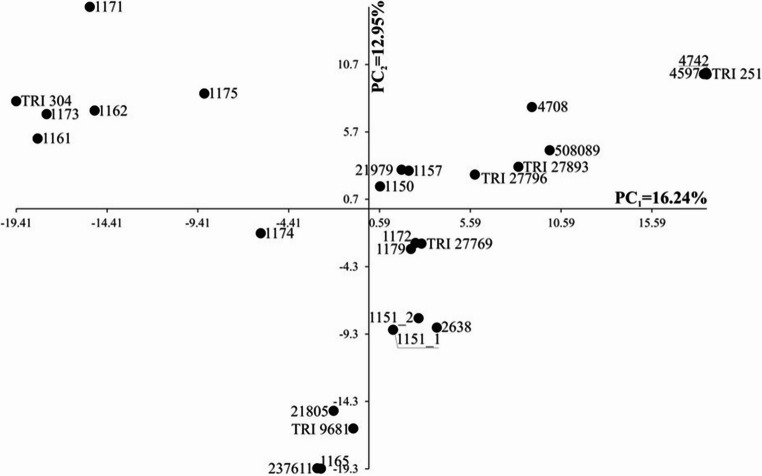
Distribution of 27 analyzed spelt wheat genotypes in the system of the first two principal components calculated based on the observations of 16,712 molecular markers

The distribution of the 27 analyzed spelt wheat genotypes in the system of the first two principal components calculated based on the observations of 16,712 molecular markers is presented in Fig. 2. The first principal component explained 16.24% of the total variability, while the second principal component explained 12.95% (Fig. 2). A division into four groups was observed. One group consisted of genotype 1174; the second group consisted of six genotypes: 1161, 1162, 1171, 1173, 1175, and TRI 304; the third group consisted of four genotypes: 1165, 21,805, 237,611, and TRI 9681; and the fourth group consisted of the remaining 16 genotypes (Fig. 2).

LD was estimated for every pairwise SilicoDArT combination within the entire germplasm collection. LD decay varied across the 21 chromosomes. LD decayed rapidly when *r*^2^ was between 0.022 and 0.05, leveling off when *r*^2^ reached 0.022. The average LD decay for the entire genome was 300 kb when *r*^2^ = 0.17.

**Fig. 3:**
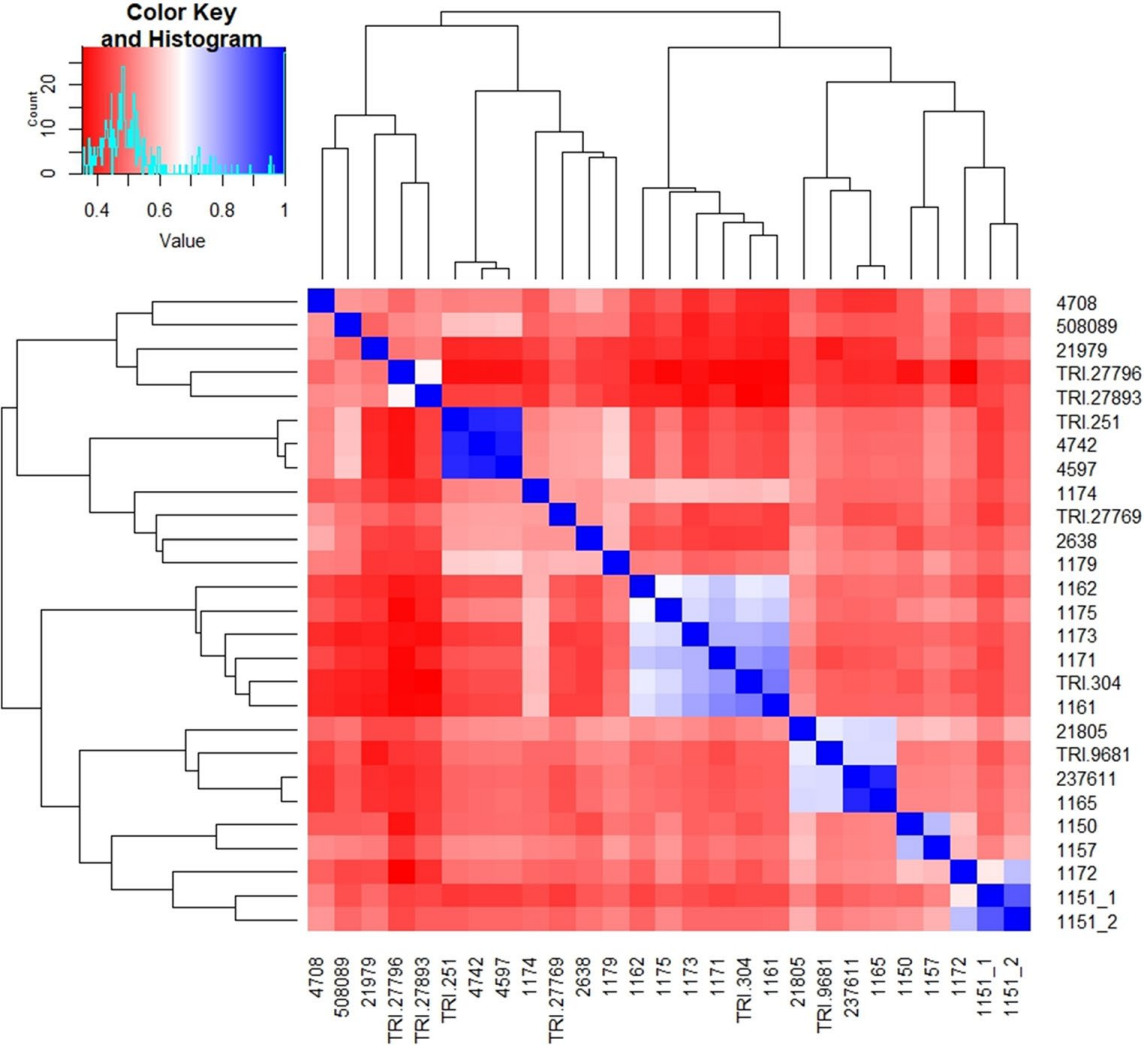
Dendrogram showing the genetic similarity between the 27 analyzed spelt wheat genotypes constructed based on the observation of 16,712 molecular markers

Illumina sequencing identified 46,813 SilicoDArT markers and 49,323 SNP markers (96136 in total). For similarity analysis and association mapping, 16,712 markers (16236 SilicoDArT and 449 SNP) meeting the criteria (MAF > 0.25 and the number of missing observations < 10%) were used. Based on these markers, the genetic similarity between all pairs of genotypes was calculated. The highest genetic similarity was observed for genotypes 4597 and 4742 (0.963), 1165 and 237,611 (0.953) as well as 4742 and TRI 251 (0.953) (Table 2). On the other hand, the highest genetic differentiation (characterized by the lowest values of genetic similarity) was observed between genotypes: 1172 and TRI 27,796 (0.351), TRI 27,893 and TRI 304 (0.351), as well as TRI 27,796 and TRI 304 (0.358) (Table 2). The calculated genetic similarity coefficients were used to construct a dendrogram of genetic similarity between the 27 studied spelt wheat genotypes. Four groups of similarity were distinguished on the dendrogram. Group I contained five genotypes (4708, 508089, 21979, TRI 27796 and TRI 27893), Group II contained seven genotypes (TRI 251, 4742, 4597, 1174, TRI 27769, 2638 and 1179), Group III contained six genotypes (1161, 1162, 1171, 1173, 1175 and TRI 304), and Group IV contained nine genotypes (1150, 1151_1, 1151_2, 1157, 1165, 1172, 21805, 237611 and TRI 9681) (Fig. 3).

**Table 2:** Genetic similarity between the 27 studied spelt wheat genotypes calculated based on 16,712 molecular markers

Genotype	1150	1151_1	1151_2	1157	1161	1162	1165	1171	1172	1173	1174	1175	1179	2638	4597	4708	4742	21,805	21,979	237,611	508,089	TRI 251	TRI 27,769	TRI 27,796	TRI 27,893	TRI 304	TRI 9681
1150	1.000																										
1151_1	0.482	1.000																									
1151_2	0.541	0.888	1.000																								
1157	0.764	0.517	0.575	1.000																							
1161	0.477	0.446	0.486	0.497	1.000																						
1162	0.486	0.437	0.478	0.510	0.714	1.000																					
1165	0.521	0.487	0.530	0.523	0.476	0.493	1.000																				
1171	0.481	0.435	0.485	0.506	0.830	0.747	0.463	1.000																			
1172	0.597	0.646	0.757	0.585	0.486	0.471	0.530	0.488	1.000																		
1173	0.475	0.451	0.484	0.486	0.791	0.711	0.475	0.776	0.470	1.000																	
1174	0.486	0.446	0.490	0.518	0.596	0.575	0.485	0.587	0.482	0.597	1.000																
1175	0.522	0.467	0.521	0.542	0.743	0.684	0.486	0.763	0.526	0.726	0.597	1.000															
1179	0.502	0.480	0.519	0.550	0.501	0.511	0.518	0.481	0.508	0.477	0.573	0.515	1.000														
2638	0.446	0.461	0.500	0.492	0.432	0.445	0.491	0.427	0.483	0.432	0.539	0.454	0.581	1.000													
4597	0.492	0.429	0.482	0.535	0.441	0.453	0.493	0.466	0.504	0.437	0.531	0.520	0.620	0.561	1.000												
4708	0.467	0.515	0.540	0.526	0.400	0.439	0.416	0.443	0.474	0.408	0.465	0.465	0.511	0.568	0.519	1.000											
4742	0.488	0.429	0.481	0.532	0.438	0.453	0.488	0.466	0.502	0.434	0.528	0.518	0.615	0.558	0.963	0.519	1.000										
21,805	0.583	0.515	0.574	0.595	0.537	0.532	0.724	0.503	0.574	0.527	0.542	0.544	0.562	0.550	0.535	0.485	0.532	1.000									
21,979	0.471	0.486	0.509	0.509	0.379	0.409	0.413	0.405	0.446	0.394	0.434	0.418	0.421	0.434	0.408	0.531	0.405	0.443	1.000								
237,611	0.518	0.480	0.523	0.519	0.473	0.491	0.953	0.460	0.527	0.471	0.482	0.481	0.511	0.488	0.487	0.412	0.483	0.720	0.410	1.000							
508,089	0.466	0.454	0.484	0.516	0.391	0.417	0.462	0.414	0.441	0.389	0.477	0.436	0.508	0.510	0.602	0.542	0.596	0.500	0.478	0.458	1.000						
TRI 251	0.484	0.420	0.471	0.526	0.428	0.446	0.479	0.456	0.493	0.424	0.518	0.504	0.610	0.552	0.948	0.513	0.953	0.523	0.401	0.478	0.594	1.000					
TRI 27,769	0.470	0.424	0.475	0.512	0.431	0.467	0.454	0.444	0.476	0.425	0.551	0.481	0.582	0.539	0.555	0.537	0.554	0.507	0.482	0.451	0.500	0.550	1.000				
TRI 27,796	0.375	0.434	0.443	0.428	0.361	0.379	0.406	0.359	0.351	0.375	0.403	0.360	0.423	0.426	0.375	0.484	0.375	0.444	0.500	0.403	0.524	0.373	0.455	1.000			
TRI 27,893	0.428	0.441	0.471	0.473	0.363	0.396	0.422	0.397	0.410	0.370	0.413	0.394	0.418	0.444	0.438	0.528	0.434	0.472	0.517	0.421	0.538	0.430	0.481	0.665	1.000		
TRI 304	0.460	0.447	0.483	0.491	0.847	0.703	0.473	0.809	0.456	0.776	0.585	0.726	0.497	0.428	0.452	0.404	0.450	0.520	0.388	0.469	0.393	0.439	0.446	0.358	0.351	1.000	
TRI 9681	0.508	0.458	0.508	0.512	0.475	0.482	0.723	0.447	0.520	0.469	0.484	0.490	0.522	0.517	0.504	0.431	0.501	0.702	0.380	0.722	0.469	0.497	0.483	0.418	0.426	0.474	1.000

**Fig. 4:**
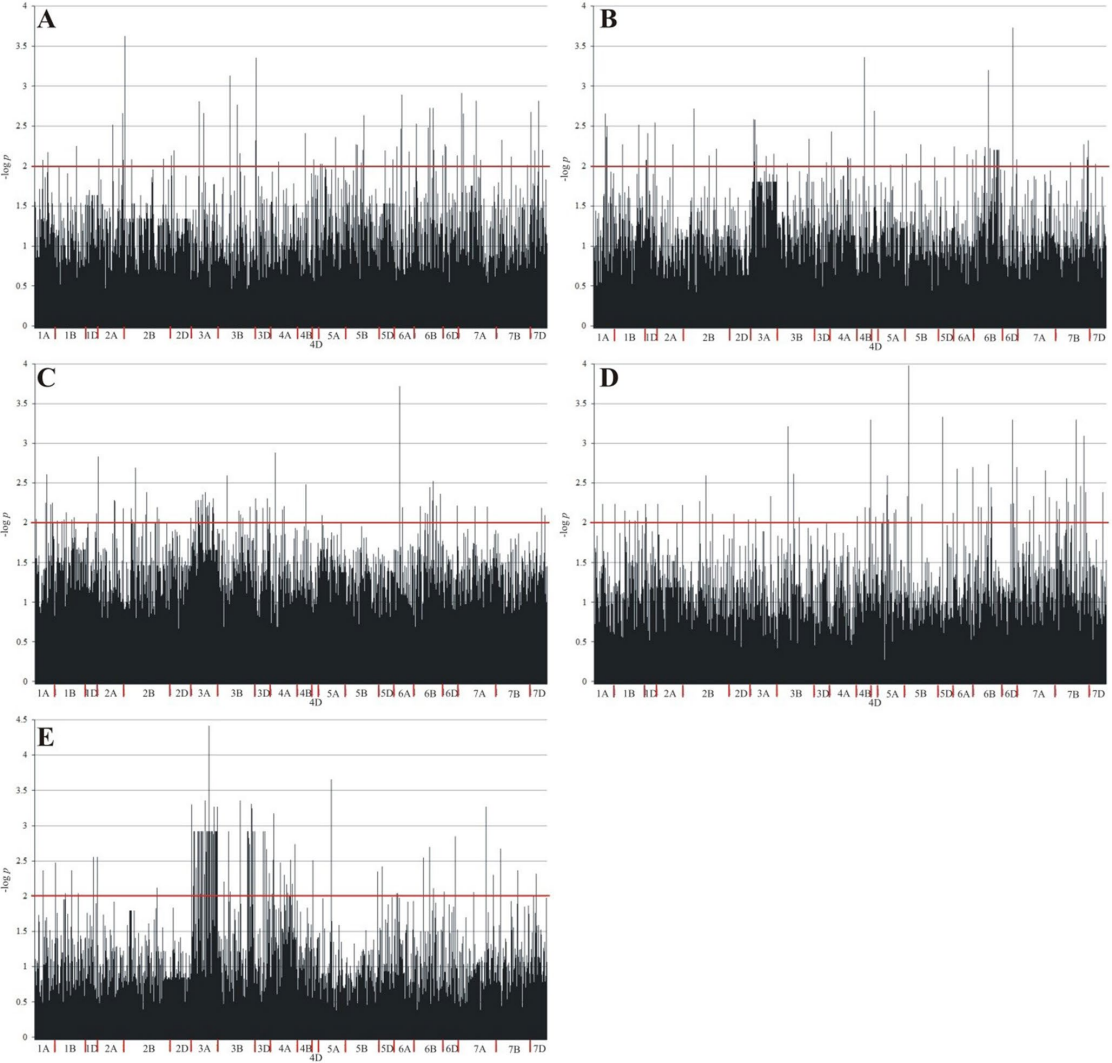
Manhattan plot for level of virulence of *B. graminis* f. sp. *tritici* for (**A**) Isolate 1, (**B**) Isolate 2, (**C**) Isolate 3, (**D**) Isolate 4, and (**E**) Isolate 5. The red line indicates LOD = 2.0, which corresponds to a significance level of 0.01

**Fig. 5:**
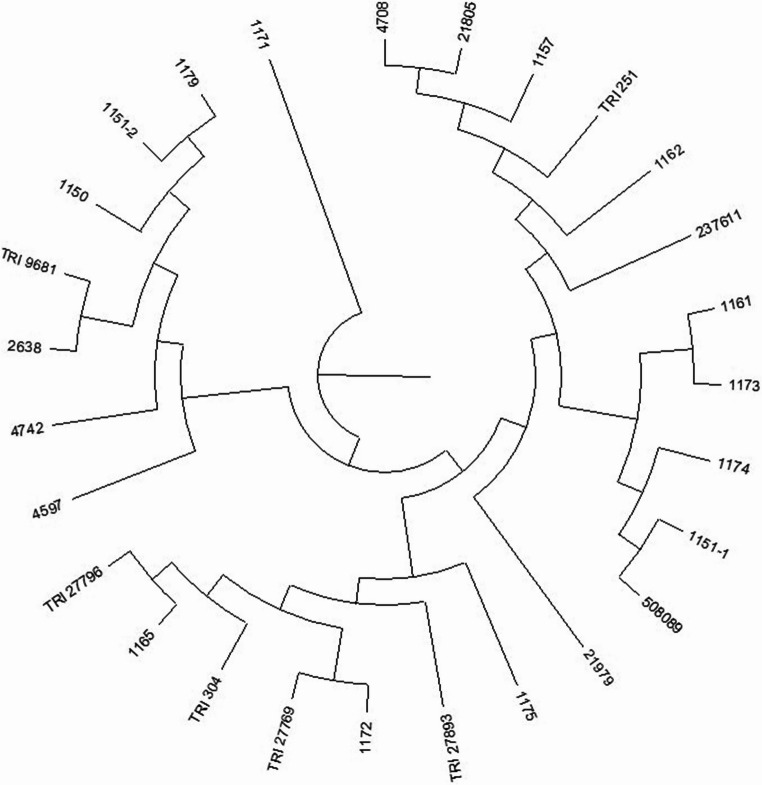
A phenotypic dendrogram based on the resistance profiles of the analyzed spelt wheat genotypes

Association mapping identified 2634 molecular markers that were significantly associated with level of virulence of *B. graminis* f. sp. *tritici* for at least one isolate at the 0.05 level (Table 3; Fig. 4). The number of markers coupled to level of virulence of *B. graminis* f. sp. *tritici* for each isolate ranged from 447 (for Isolate5) to 1172 (for Isolate3) (Table 3; Fig. 4). The percentage of variation explained by each marker ranged from 11.1% to 10.1% for Isolate 1, from 11.1% to 41.1% for Isolate2 and Isolate 3, from 11.1% to 43.7% for Isolate4 and from 11.1% to 47.9% for Isolate5 (Table 3). None of the markers were statistically significant for all five isolates. Only one marker (SilicoDArT 1102744, located on chromosome 3D) significantly determined the level of virulence of *B. graminis* f. sp. *tritici* for four of the five isolates (except Isolate1). 110 markers (70 SilicoDArT and 40 SNP) had a statistically significant effect on the observed trait for three of five isolates. Of the selected markers, 366 determined the level of virulence of *B. graminis* f. sp. *tritici* at 0.01 (Table S1; Fig. 4). Two of these markers (SilicoDArT 7492586 and SNP 1126088|F|0–9:C > T-9:C > T**)** determined the virulence level for three of the five isolates: Isolate1, Isolate2, and Isolate5 (Table S1). The percentage of variability explained by these two markers was substantial: for SilicoDArT 7,492,586 it was 23.5% (Isolate2), 23.2% (Isolate3), and 22.3% (Isolate5); for SNP 1,126,088 it was 25.1% (Isolate2), 23.3% (Isolate3), and 30.1% (Isolate5) (Table S1). Some markers exhibit positive effects (increasing resistance) and others negative effects (promoting susceptibility), reflecting the complex genetic nature of powdery mildew resistance in spelt wheat. For example, marker SilicoDArT 4,397,580 on chromosome 1 A exhibits a high positive effect (Estimate = 72.8) on Isolate2, while marker 2,254,313 on the same chromosome exhibits a negative effect (−69.3) on Isolate2, suggesting the presence of different resistance genes or alleles with opposing effects. Many resistance-associated markers are concentrated on chromosomes 1A, 3 A, 6B, and 7 A, which may indicate the presence of resistance-related QTLs (Quantitative Trait Loci) in these regions of the genome. On some chromosomes, such as 3 A and 7B, markers with both large positive and negative effects appear, suggesting differential genotype-pathogen isolate interactions. Some markers exhibit high LOD and Proc values only for a specific isolate. For example, SNP 1,077,141 on chromosome 3 A has a very high LOD (4.42) and explains a large proportion of the variance (47.9%) for Isolate3 but has no significant effect for the other isolates. This indicates that resistance is not universal but rather strain-specific (Fig. 5).

**Table 3:** Characterization of the significance of molecular markers determining level of virulence of *B*. *graminis* f. sp. *tritici* for each isolate (significant associations selected at *p* < 0.05 with correction for Benjamini–Hochberg multiple testing)

Isolate	Marker type	All markers		SilicoDArT		SNP	
		min	max	min	max	min	max
Isolate1	Number of significant markers	515		380		135	
	Estimate	−43.4	41.1	−43.4	41.1	−37.3	35
	Percentage variance accounted for	11.1	40.1	11.1	40.1	11.1	29.1
	LOD	1.301	3.630	1.305	3.630	1.301	2.662
Isolate2	Number of significant markers	470		325		145	
	Estimate	−60.8	50.1	−60.8	50.1	−49.3	49.3
	Percentage variance accounted for	11.1	41.1	11.1	41.1	11.1	27.6
	LOD	1.301	3.729	1.301	3.729	1.304	2.543
Isolate3	Number of significant markers	1172		787		385	
	Estimate	−76.1	92.5	−76.1	92.5	−72.9	72.8
	Percentage variance accounted for	11.1	41.1	11.1	41.1	11.1	31.2
	LOD	1.301	3.723	1.301	3.723	1.303	2.833
Isolate4	Number of significant markers	461		341		120	
	Estimate	−69.2	61.2	−69.2	61.2	−68.7	56.7
	Percentage variance accounted for	11.1	43.7	11.1	43.7	11.1	36.6
	LOD	1.302	3.981	1.302	3.981	1.302	3.303
Isolate5	Number of significant markers	447		304		143	
	Estimate	−70	64.4	−67.4	64.4	−70	58.8
	Percentage variance accounted for	11.1	47.9	11.1	40.4	11.1	47.9
	LOD	1.303	4.419	1.305	3.658	1.303	4.419

## Discussion

The depletion of the available resistance gene pool in modern cereal varieties has prompted scientists and breeders to search for new, effective sources of resistance, including among ancient cereal species such as spelt (Hajjar and Hodgkin 2007; Feuillet et al. 2008). Due to the key role of cereals in global food security and the changing climate, research is intensifying on the identification of genotypes resistant to cereal diseases, including powdery mildew of cereals and grasses. A similar approach has been successfully applied to wild wheat relatives, such as *Aegilops biuncialis*. In this case, GWAS combined with DArTseq genotyping identified 34 QTLs associated with fiber and protein content (Ivanizs et al. 2022). This demonstrates the potential of using wild relatives and modern genomic tools to discover loci linked to valuable traits, including disease resistance. The study evaluated 86 genotypes from 16 countries, representing diverse ecological and geographical backgrounds. High-density genotyping with 32,700 SilicoDArT markers enabled detailed analysis of genetic diversity and population structure. This provides a solid reference for comparative genomic studies. (Ivanizs et al. 2019). The pathogen *B. graminis* f. sp. *tritici* shows high adaptability. Its virulence variability positively correlates with the prevalence of varieties containing specific resistance genes (de Vallavieille-Pope et al. 2000; Zhang et al. 2022). Therefore, our integrated approach to resistance assessment of spelt wheat genotypes allowed us to identify the differential resistance response to powdery mildew of cereals and grasses induced by five differentiating Bgt isolates.

The virulence analysis showed that Isolate3 and Isolate5 were characterized by the highest pathogenicity, as confirmed by both high mean infection values and wide range of variability. In contrast, Isolate2 showed the lowest virulence. We believe that this may indicate the presence of effective resistance mechanisms in some spelt wheat lines, such as 1151_1, 500,089 and TRI 2798. These results are in line with the findings of McDonald and Linde (2002), who emphasize the dynamic evolution of pathogens under the selective pressure of host plants. A key factor influencing the durability of plant resistance is the evolutionary potential of pathogens, which depends on their genetic structure (McDonald and Linde 2002).

Dendrogram and heat map analysis revealed the existence of four main groups of spelt genotypes. They include both pairs with a high degree of similarity (4708 and 4742) and clearly different genotypes (TRI 27796, TRI 27893). The high coefficient of genetic similarity (0.89–0.96) observed between some lines may suggest their potential duplication. At the same time, principal component analysis (PCA) revealed that the first component (PC1) explains half of the total phenotypic variance, and the extreme positions of genotypes such as TRI 27,893 and 4597 may indicate their different resistance profile. The phenotypic dendrogram confirmed the existence of both genotype groups with a high degree of similarity (e.g. 1151_1, 500089, TRI 2798) and significantly differentiated lines (e.g. TRI 251, 4708, 21805).

Among the analyzed Bgt isolates, Isolate3 and Isolate5 stood out in particular. The former was characterized by the largest number of significant markers (84), which may indicate its high genetic variability and complex interactions with various wheat genotypes. In turn, Isolate5, despite a smaller number of associated markers (27), achieved the highest LOD values (up to 9.43), which suggests the presence of strong virulence loci with a large impact. Similar observations were made by Simeone et al. (2020), identifying QTL associated with resistance in tetraploid wheat. Heatmap analysis and isolate clustering allowed us to distinguish three virulence groups: (1) Isolate3 and Isolate5 – the most pathogenic, (2) Isolate1 and Isolate4 – moderately virulent, and (3) Isolate2 – showing the lowest infectious potential. The diversity of pathogen responses may be the result not only of genotypic differences between isolates, but also of internal phenotypic and epigenetic variability of host plants, which indicates the complex, multi-level nature of host-pathogen interactions. As emphasized by Sorbal and Sampedro (2022), sub-individual plant variability may play an important role in the course and direction of pathogen evolution, shaping their virulence in a dynamic and environmentally dependent manner.

In the analysis of pathogen isolates, significant differences were observed in both the number and type of detected molecular markers, which reflects the high genetic variability of the pathogen *B. graminis* f. sp. *tritici*. This variability directly affects the phenotypic response of plants, emphasizing the dynamic and variable nature of plant–pathogen interactions. Similar findings were presented by Hurni et al. (2014), indicating that some resistance genes, such as *Pm*3 and *Pm*8, retain the ability to recognize the pathogen despite large evolutionary distances. In turn, Liu et al. (2022) identified QTLs associated with powdery mildew resistance on chromosomes 2B and 5B, and our results also confirm the importance of chromosome 6 A, previously indicated by Wang et al. (2024) as a key region responsible for resistance to *B. graminis* f. sp. *tritici*.

The comparison of marker types used in genetic analyses revealed that SilicoDArT markers were more numerous and showed a wider range of effect values, which may indicate their greater sensitivity to genomic variability Similar applications of DArTseq have been reported for wild wheat relatives, such as Aegilops biuncialis (Ivanizs et al. 2019, 2022). In that study, SilicoDArT markers were used to assess genetic diversity and population structure in 86 genotypes. The analysis revealed five subpopulations that correlated with ecological and geographical origin. These results show that DArTseq can provide valuable insights even in small collections (< 100 genotypes). This approach also allows linking genetic structure with adaptive traits, such as heading time.In turn, SNP markers, although characterized by a lower polymorphism index value (average PIC ≈ 0.165) due to their bi-allelic nature, showed greater stability and repeatability. Stable SNPs are particularly useful in marker-assisted selection (MAS), especially in the context of local varieties and wild species (Baloch et al. 2017). A similar approach, based on DArTseq technology, was used in studies of durum wheat (Robbana et al. 2019), which allowed for precise determination of the population structure and assessment of the breeding potential of local varieties. In these analyses, similarly to our studies, the use of both SilicoDArT and SNP markers allowed for obtaining complementary information on genomic variability. SilicoDArT markers proved to be particularly useful in analyses of population structure, while SNP markers were effective in differentiating population structures and valuable in association and MAS studies (Robbana et al. 2019; Ebrahimi et al. 2025). Their combined use enables comprehensive characterization of the genome and identification of loci associated with adaptive traits, important in the context of environmental stress.

In the present study, SilicoDArT and SNP markers were successfully used to identify regions associated with resistance. Particularly significant signals were obtained on chromosomes 3D, 5B and 6 A – whereby chromosome 3D may contain broad-spectrum resistance genes, chromosome 5B (LOD > 4.1) may act as a QTL “hotspot”, while chromosome 6 A showed strong and reproducible signals for the four analyzed isolates.

The obtained results confirm the polygenic nature of powdery mildew resistance. Genes such as *Pm*2 (2 A), *Pm*3 (1 A), *Pm*4b (2 A), *Pm*8 (1B) and *Pm*21 (6 V) have been previously indicated as important sources of resistance (Bhullar et al. 2010; Mohler et al. 2010; Ma et al. 2016). Importantly, the marker signals located on the 3D chromosome in our study overlap with the locations of QTL associated with adult plant resistance (APR) described by Zeng et al. (2014) and Singh et al. (2008), which additionally strengthens the credibility of the obtained results and indicates the possibility of their practical use in breeding programs.

In summary, the conducted studies provided valuable information on the molecular basis of spelt resistance to *B. graminis* f. sp. *tritici*. DArTseq technology enabled the identification of numerous markers associated with the phenotypic response to infection, confirming the high genetic variability both within wheat genotypes and pathogen isolates. Markers showing stable and strong associations with resistance are a valuable resource for future breeding programs, especially in the context of the growing interest in spelt as an organic cereal, where the use of chemical plant protection products is limited. The identified chromosomal regions (1D, 3D, 5B, 6 A) require further exploration, and the genetic diversity of the collection provides a solid basis for the development of varieties resistant to biotic stresses. Although the identification of markers is an important step, further functional studies, such as analysis of gene expression in response to infection, are necessary to provide a more complete understanding of the resistance mechanisms and to confirm the role of the indicated loci.

## Supplementary Information

Below is the link to the electronic supplementary material.

ESM 1 (DOCX 114 KB)
