# Supplementary material for: Characterization of spelt wheat (Triticum spelta L.) genotypes using DArTseq technology
Source: J Appl Genet. 2025 Dec 27;67(2):285–99. doi: 10.1007/s13353-025-01037-4 (PMC13079525; doi:10.1007/s13353-025-01037-4)
Supplement: Supplementary file 1 — (DOCX 114 KB) [file 13353_2025_1037_MOESM1_ESM.docx]

Table S1. Markers statistically significantly (p<0.01) associated with the observed trait for individual isolates

| Chr | Marker type | CloneID | Izolate 1 | | | Izolate 2 | | | Izolate 3 | | | Izolate 4 | | | Izolate 5 | | |
| --- | --- | --- | --- | --- | --- | --- | --- | --- | --- | --- | --- | --- | --- | --- | --- | --- | --- |
|  |  |  | Estimate | Proc | LOD | Estimate | Proc | LOD | Estimate | Proc | LOD | Estimate | Proc | LOD | Estimate | Proc | LOD |
| 1A | SilicoDArT | 1132186 |  |  |  |  |  |  | 62.9 | 21.3 | 2.05 |  |  |  |  |  |  |
| 1A | SilicoDArT | 1105032 |  |  |  |  |  |  |  |  |  |  |  |  | -53.6 | 25.5 | 2.37 |
| 1A | SilicoDArT | 3954215 | 36.7 | 21.7 | 2.08 |  |  |  |  |  |  |  |  |  |  |  |  |
| 1A | SilicoDArT | 987095 |  |  |  |  |  |  |  |  |  | -54.5 | 23.7 | 2.24 |  |  |  |
| 1A | SilicoDArT | 2254313 |  |  |  |  |  |  | -69.3 | 23.4 | 2.21 |  |  |  |  |  |  |
| 1A | SilicoDArT | 4397580 |  |  |  |  |  |  | 72.8 | 23.9 | 2.25 |  |  |  |  |  |  |
| 1A | SilicoDArT | 1102766 |  |  |  | -47.8 | 29 | 2.65 | -69.2 | 28.4 | 2.61 |  |  |  |  |  |  |
| 1A | SilicoDArT | 1053692 |  |  |  | -46.4 | 25.4 | 2.36 |  |  |  |  |  |  |  |  |  |
| 1A | SilicoDArT | 1219904 |  |  |  | -47.7 | 27.1 | 2.50 |  |  |  |  |  |  |  |  |  |
| 1A | SilicoDArT | 4910422 | -35 | 23 | 2.18 |  |  |  |  |  |  |  |  |  |  |  |  |
| 1A | SNP | 3064401\|F\|0-7:C>G-7:C>G |  |  |  |  |  |  | 67.5 | 23.7 | 2.23 |  |  |  |  |  |  |
| 1A | SNP | 2254313\|F\|0-7:T>A-7:T>A |  |  |  |  |  |  | 72.8 | 23.9 | 2.25 |  |  |  |  |  |  |
| 1A | SNP | 1002571\|F\|0-5:C>G-5:C>G |  |  |  |  |  |  |  |  |  |  |  |  | -53.3 | 26.8 | 2.47 |
| 1A | SNP | 992499\|F\|0-48:A>G-48:A>G |  |  |  |  |  |  |  |  |  | -56.8 | 23.7 | 2.23 |  |  |  |
| 1B | SilicoDArT | 3384860 |  |  |  |  |  |  | 60.8 | 21 | 2.03 |  |  |  |  |  |  |
| 1B | SilicoDArT | 1219650 |  |  |  |  |  |  | -60.3 | 20.9 | 2.02 |  |  |  |  |  |  |
| 1B | SilicoDArT | 1099463 |  |  |  |  |  |  | -61.7 | 21.2 | 2.04 |  |  |  |  |  |  |
| 1B | SilicoDArT | 1080144 |  |  |  | 50.1 | 24.3 | 2.28 |  |  |  |  |  |  |  |  |  |
| 1B | SilicoDArT | 2275932 |  |  |  |  |  |  |  |  |  | 55.7 | 22.7 | 2.15 | 54.7 | 21.2 | 2.04 |
| 1B | SilicoDArT | 4260938 |  |  |  |  |  |  |  |  |  | -55.7 | 22.7 | 2.15 | -54.7 | 21.2 | 2.04 |
| 1B | SilicoDArT | 39605077 |  |  |  |  |  |  | 70.8 | 22.4 | 2.13 |  |  |  |  |  |  |
| 1B | SilicoDArT | 3222538 |  |  |  |  |  |  |  |  |  | -49 | 21.1 | 2.03 |  |  |  |
| 1B | SilicoDArT | 1129852 |  |  |  |  |  |  |  |  |  |  |  |  | -53.6 | 25.5 | 2.37 |
| 1B | SilicoDArT | 1219536 |  |  |  |  |  |  | -61.7 | 21.2 | 2.04 |  |  |  |  |  |  |
| 1B | SilicoDArT | 1022144 |  |  |  |  |  |  | -62.2 | 21.6 | 2.07 |  |  |  |  |  |  |
| 1B | SilicoDArT | 4407754 |  |  |  |  |  |  |  |  |  | 51.8 | 21 | 2.03 |  |  |  |
| 1B | SilicoDArT | 990280 | 34.8 | 24 | 2.25 |  |  |  |  |  |  |  |  |  |  |  |  |
| 1B | SNP | 2275932\|F\|0-39:A>T-39:A>T |  |  |  |  |  |  |  |  |  | -55.7 | 22.7 | 2.15 | -54.7 | 21.2 | 2.04 |
| 1B | SNP | 1263607\|F\|0-11:G>T-11:G>T |  |  |  | -49.1 | 27.3 | 2.52 |  |  |  |  |  |  |  |  |  |
| 1B | SNP | 1051533\|F\|0-5:G>A-5:G>A |  |  |  |  |  |  |  |  |  | -56.8 | 23.7 | 2.23 |  |  |  |
| 1D | SilicoDArT | 1217937 |  |  |  |  |  |  |  |  |  | 50.7 | 21.5 | 2.07 |  |  |  |
| 1D | SilicoDArT | 2255418 |  |  |  | 42.6 | 21.7 | 2.08 |  |  |  |  |  |  |  |  |  |
| 1D | SilicoDArT | 5332971 |  |  |  |  |  |  | 60.1 | 20.7 | 2.00 |  |  |  |  |  |  |
| 1D | SilicoDArT | 1100775 |  |  |  | -45.9 | 21.7 | 2.08 |  |  |  |  |  |  |  |  |  |
| 1D | SilicoDArT | 1159433 |  |  |  | -45.6 | 26 | 2.41 |  |  |  |  |  |  |  |  |  |
| 1D | SilicoDArT | 100050201 |  |  |  |  |  |  |  |  |  |  |  |  | -55.7 | 27.8 | 2.56 |
| 1D | SNP | 2252023\|F\|0-38:C>T-38:C>T |  |  |  | -46.5 | 27.6 | 2.54 | -61.9 | 22.2 | 2.12 |  |  |  |  |  |  |
| 1D | SNP | 2255388\|F\|0-16:G>A-16:G>A |  |  |  |  |  |  |  |  |  |  |  |  | -55.7 | 27.8 | 2.56 |
| 1D | SNP | 3937379\|F\|0-6:G>A-6:G>A |  |  |  |  |  |  | -72.9 | 31.2 | 2.83 |  |  |  |  |  |  |
| 1D | SNP | 1030253\|F\|0-27:T>C-27:T>C |  |  |  |  |  |  |  |  |  | -56.8 | 23.7 | 2.23 |  |  |  |
| 2A | SilicoDArT | 1099938 | 32.9 | 21.9 | 2.09 |  |  |  |  |  |  |  |  |  |  |  |  |
| 2A | SilicoDArT | 4002509 | -40.5 | 27.3 | 2.52 |  |  |  |  |  |  |  |  |  |  |  |  |
| 2A | SilicoDArT | 1377958 |  |  |  | -45.4 | 24.2 | 2.27 | -66.7 | 24.4 | 2.29 |  |  |  |  |  |  |
| 2A | SilicoDArT | 4406798 |  |  |  |  |  |  | -64.2 | 24.2 | 2.27 |  |  |  |  |  |  |
| 2A | SNP | 4009346\|F\|0-34:C>T-34:C>T |  |  |  |  |  |  | 71.7 | 23 | 2.18 |  |  |  |  |  |  |
| 2A | SNP | 1022654\|F\|0-66:T>C-66:T>C | -36.6 | 29.1 | 2.66 |  |  |  |  |  |  |  |  |  |  |  |  |
| 2A | SNP | 1051506\|F\|0-21:A>T-21:A>T |  |  |  |  |  |  |  |  |  | 56.7 | 23.6 | 2.22 |  |  |  |
| 2A | SNP | 1257186\|F\|0-15:T>C-15:T>C | -33.3 | 21.7 | 2.08 |  |  |  |  |  |  |  |  |  |  |  |  |
| 2B | SilicoDArT | 1213706 | -42.9 | 40.1 | 3.63 |  |  |  |  |  |  |  |  |  |  |  |  |
| 2B | SilicoDArT | 1084021 |  |  |  |  |  |  | -63.2 | 23 | 2.18 |  |  |  |  |  |  |
| 2B | SilicoDArT | 1233120 |  |  |  |  |  |  | -61.7 | 21.2 | 2.04 |  |  |  |  |  |  |
| 2B | SilicoDArT | 1165797 | -34.2 | 21.7 | 2.08 |  |  |  |  |  |  |  |  |  |  |  |  |
| 2B | SilicoDArT | 5332907 |  |  |  | 48.9 | 29.8 | 2.72 | 71.1 | 29.5 | 2.70 |  |  |  |  |  |  |
| 2B | SilicoDArT | 1103433 |  |  |  |  |  |  |  |  |  | -55 | 24.2 | 2.27 |  |  |  |
| 2B | SilicoDArT | 3533595 |  |  |  |  |  |  | -67.4 | 22 | 2.10 |  |  |  |  |  |  |
| 2B | SilicoDArT | 3533674 |  |  |  |  |  |  | 68.1 | 25.6 | 2.38 |  |  |  |  |  |  |
| 2B | SilicoDArT | 4990329 |  |  |  |  |  |  |  |  |  | 55.6 | 28.3 | 2.60 |  |  |  |
| 2B | SilicoDArT | 5324205 |  |  |  | -46.5 | 22.4 | 2.13 |  |  |  |  |  |  |  |  |  |
| 2B | SilicoDArT | 4536336 |  |  |  |  |  |  |  |  |  | -55.1 | 22.1 | 2.11 |  |  |  |
| 2B | SilicoDArT | 7492586 |  |  |  | 46 | 23.5 | 2.22 | 66.8 | 23.2 | 2.19 |  |  |  | 51.9 | 22.3 | 2.12 |
| 2B | SNP | 68425911\|F\|0-26:G>A-26:G>A |  |  |  |  |  |  | -62.9 | 21.3 | 2.05 |  |  |  |  |  |  |
| 2B | SNP | 979702\|F\|0-10:C>G-10:C>G | -34.2 | 21.8 | 2.09 |  |  |  |  |  |  |  |  |  |  |  |  |
| 2D | SilicoDArT | 2323768 | -37.2 | 22.4 | 2.13 |  |  |  |  |  |  |  |  |  |  |  |  |
| 2D | SilicoDArT | 2282310 | 36.3 | 23.2 | 2.19 |  |  |  |  |  |  |  |  |  |  |  |  |
| 2D | SilicoDArT | 7491744 |  |  |  |  |  |  |  |  |  | -50 | 22.1 | 2.11 |  |  |  |
| 2D | SNP | 5366796\|F\|0-5:A>C-5:A>C |  |  |  |  |  |  |  |  |  | 48.3 | 21.2 | 2.04 |  |  |  |
| 2D | SNP | 1065249\|F\|0-25:A>G-25:A>G |  |  |  |  |  |  | 62.1 | 21.5 | 2.06 |  |  |  |  |  |  |
| 3A | SilicoDArT | 1204275 |  |  |  |  |  |  |  |  |  |  |  |  | 64.4 | 36.5 | 3.30 |
| 3A | SilicoDArT | 4409709 |  |  |  |  |  |  |  |  |  |  |  |  | 49.5 | 22.6 | 2.15 |
| 3A | SilicoDArT | 3960674 |  |  |  |  |  |  |  |  |  |  |  |  | 58.4 | 32.2 | 2.92 |
| 3A | SilicoDArT | 3937102 |  |  |  |  |  |  |  |  |  |  |  |  | 49.5 | 22.6 | 2.15 |
| 3A | SilicoDArT | 1105981 |  |  |  |  |  |  |  |  |  |  |  |  | 58.4 | 32.2 | 2.92 |
| 3A | SilicoDArT | 4538283 |  |  |  |  |  |  |  |  |  |  |  |  | -60.6 | 27 | 2.49 |
| 3A | SilicoDArT | 1120795 |  |  |  |  |  |  |  |  |  |  |  |  | -51.3 | 21.7 | 2.08 |
| 3A | SilicoDArT | 100000061 |  |  |  | -47.7 | 28.2 | 2.59 |  |  |  |  |  |  |  |  |  |
| 3A | SilicoDArT | 1255457 |  |  |  | -41.9 | 21.4 | 2.06 |  |  |  |  |  |  |  |  |  |
| 3A | SilicoDArT | 4004924 |  |  |  |  |  |  | 69.2 | 23.4 | 2.21 |  |  |  |  |  |  |
| 3A | SilicoDArT | 4538631 |  |  |  | -49.7 | 28.1 | 2.58 | -68.2 | 24.3 | 2.28 |  |  |  |  |  |  |
| 3A | SilicoDArT | 1089657 |  |  |  |  |  |  |  |  |  |  |  |  | -59.5 | 25.9 | 2.40 |
| 3A | SilicoDArT | 1202831 |  |  |  |  |  |  |  |  |  | -47.6 | 21.3 | 2.05 |  |  |  |
| 3A | SilicoDArT | 1379114 |  |  |  | -45.4 | 24.2 | 2.27 | -66.7 | 24.4 | 2.29 |  |  |  |  |  |  |
| 3A | SilicoDArT | 1082727 |  |  |  |  |  |  |  |  |  |  |  |  | -58.4 | 32.2 | 2.92 |
| 3A | SilicoDArT | 1298428 |  |  |  |  |  |  |  |  |  |  |  |  | -58.4 | 32.2 | 2.92 |
| 3A | SilicoDArT | 1119840 |  |  |  |  |  |  |  |  |  |  |  |  | -58.4 | 32.2 | 2.92 |
| 3A | SilicoDArT | 2266162 |  |  |  |  |  |  |  |  |  |  |  |  | -58.4 | 32.2 | 2.92 |
| 3A | SilicoDArT | 3937797 |  |  |  |  |  |  |  |  |  |  |  |  | -55.7 | 27.7 | 2.55 |
| 3A | SilicoDArT | 39465726 |  |  |  |  |  |  |  |  |  |  |  |  | -58.4 | 32.2 | 2.92 |
| 3A | SilicoDArT | 1114897 |  |  |  |  |  |  |  |  |  |  |  |  | -58.4 | 32.2 | 2.92 |
| 3A | SilicoDArT | 1279653 |  |  |  |  |  |  | 69 | 23.2 | 2.19 |  |  |  |  |  |  |
| 3A | SilicoDArT | 1138471 |  |  |  |  |  |  | -63.9 | 20.8 | 2.01 |  |  |  |  |  |  |
| 3A | SilicoDArT | 1100015 | -37.8 | 30.9 | 2.81 |  |  |  |  |  |  |  |  |  |  |  |  |
| 3A | SilicoDArT | 1125772 |  |  |  |  |  |  |  |  |  |  |  |  | -58.4 | 32.2 | 2.92 |
| 3A | SilicoDArT | 1090449 |  |  |  |  |  |  |  |  |  |  |  |  | 58.4 | 32.2 | 2.92 |
| 3A | SilicoDArT | 1096288 |  |  |  |  |  |  |  |  |  |  |  |  | 58.4 | 32.2 | 2.92 |
| 3A | SilicoDArT | 1254515 |  |  |  |  |  |  |  |  |  |  |  |  | -52.4 | 21.1 | 2.04 |
| 3A | SilicoDArT | 2245014 |  |  |  |  |  |  | -64.5 | 24.4 | 2.29 |  |  |  |  |  |  |
| 3A | SilicoDArT | 5000814 |  |  |  |  |  |  |  |  |  |  |  |  | -62.7 | 29.1 | 2.67 |
| 3A | SilicoDArT | 1105410 |  |  |  |  |  |  |  |  |  |  |  |  | -58.4 | 32.2 | 2.92 |
| 3A | SilicoDArT | 5323445 |  |  |  |  |  |  |  |  |  |  |  |  | -53.5 | 27 | 2.49 |
| 3A | SilicoDArT | 3939101 |  |  |  |  |  |  |  |  |  |  |  |  | 58.4 | 32.2 | 2.92 |
| 3A | SilicoDArT | 1200653 |  |  |  |  |  |  |  |  |  |  |  |  | 52.3 | 25.6 | 2.38 |
| 3A | SilicoDArT | 3946963 |  |  |  |  |  |  |  |  |  |  |  |  | -58.4 | 32.2 | 2.92 |
| 3A | SilicoDArT | 1078744 |  |  |  |  |  |  |  |  |  |  |  |  | 51.8 | 25.1 | 2.34 |
| 3A | SilicoDArT | 1064863 |  |  |  |  |  |  |  |  |  |  |  |  | 55.7 | 27.7 | 2.55 |
| 3A | SilicoDArT | 4541224 |  |  |  |  |  |  | 61.6 | 22 | 2.10 |  |  |  |  |  |  |
| 3A | SilicoDArT | 5324968 |  |  |  |  |  |  | -62.7 | 22 | 2.10 |  |  |  |  |  |  |
| 3A | SilicoDArT | 3940860 |  |  |  |  |  |  | -66.5 | 25.3 | 2.36 |  |  |  |  |  |  |
| 3A | SilicoDArT | 3533335 |  |  |  |  |  |  |  |  |  |  |  |  | -58.4 | 32.2 | 2.92 |
| 3A | SilicoDArT | 5339736 |  |  |  |  |  |  |  |  |  |  |  |  | -58.4 | 32.2 | 2.92 |
| 3A | SilicoDArT | 1093895 |  |  |  |  |  |  |  |  |  |  |  |  | 51.8 | 25.5 | 2.37 |
| 3A | SilicoDArT | 2253031 |  |  |  |  |  |  |  |  |  |  |  |  | 58.4 | 32.2 | 2.92 |
| 3A | SilicoDArT | 2275850 |  |  |  |  |  |  |  |  |  |  |  |  | 48.8 | 21.3 | 2.05 |
| 3A | SilicoDArT | 4910486 | -36.8 | 29.1 | 2.66 |  |  |  |  |  |  |  |  |  |  |  |  |
| 3A | SilicoDArT | 7331406 |  |  |  |  |  |  |  |  |  |  |  |  | -54.2 | 24.7 | 2.31 |
| 3A | SilicoDArT | 1228184 |  |  |  |  |  |  | -72.1 | 25.7 | 2.39 |  |  |  |  |  |  |
| 3A | SilicoDArT | 6045039 |  |  |  |  |  |  | -63.8 | 22 | 2.10 |  |  |  | -63.4 | 37.2 | 3.36 |
| 3A | SilicoDArT | 4539802 |  |  |  |  |  |  | 63 | 23.2 | 2.19 |  |  |  |  |  |  |
| 3A | SilicoDArT | 1140908 |  |  |  |  |  |  |  |  |  |  |  |  | -55.5 | 28.7 | 2.63 |
| 3A | SilicoDArT | 4990694 |  |  |  |  |  |  |  |  |  |  |  |  | -58.4 | 32.2 | 2.92 |
| 3A | SilicoDArT | 1024193 |  |  |  |  |  |  | -63.3 | 23.5 | 2.21 |  |  |  |  |  |  |
| 3A | SilicoDArT | 4990704 |  |  |  |  |  |  | -62.2 | 22.5 | 2.14 |  |  |  |  |  |  |
| 3A | SilicoDArT | 1114248 |  |  |  |  |  |  |  |  |  |  |  |  | -58.4 | 32.2 | 2.92 |
| 3A | SilicoDArT | 4539630 |  |  |  |  |  |  | 60.1 | 20.7 | 2.00 |  |  |  | 55.4 | 29.7 | 2.71 |
| 3A | SilicoDArT | 992268 |  |  |  | 42.7 | 22.3 | 2.13 |  |  |  |  |  |  | 56.5 | 30.6 | 2.79 |
| 3A | SilicoDArT | 1237234 |  |  |  |  |  |  |  |  |  |  |  |  | -58.4 | 32.2 | 2.92 |
| 3A | SilicoDArT | 62177765 |  |  |  |  |  |  | -61.8 | 21.9 | 2.09 |  |  |  |  |  |  |
| 3A | SilicoDArT | 3534373 |  |  |  |  |  |  |  |  |  |  |  |  | 58.4 | 32.2 | 2.92 |
| 3A | SNP | 1077141\|F\|0-25:C>G-25:C>G |  |  |  |  |  |  |  |  |  |  |  |  | -70 | 47.9 | 4.42 |
| 3A | SNP | 1081242\|F\|0-25:A>G-25:A>G |  |  |  |  |  |  |  |  |  |  |  |  | -62.7 | 29.1 | 2.67 |
| 3A | SNP | 1091514\|F\|0-56:A>T-56:A>T |  |  |  |  |  |  | -62.8 | 22.1 | 2.11 |  |  |  |  |  |  |
| 3A | SNP | 2257330\|F\|0-24:C>T-24:C>T |  |  |  |  |  |  | -64 | 24.1 | 2.26 |  |  |  |  |  |  |
| 3A | SNP | 1114897\|F\|0-28:C>A-28:C>A |  |  |  |  |  |  |  |  |  |  |  |  | 58.4 | 32.2 | 2.92 |
| 3A | SNP | 2261992\|F\|0-67:A>G-67:A>G |  |  |  |  |  |  |  |  |  |  |  |  | -58.4 | 32.2 | 2.92 |
| 3A | SNP | 1179384\|F\|0-51:T>G-51:T>G |  |  |  |  |  |  |  |  |  |  |  |  | -62.7 | 29.1 | 2.67 |
| 3A | SNP | 994102\|F\|0-54:G>A-54:G>A |  |  |  |  |  |  |  |  |  |  |  |  | -55.7 | 27.7 | 2.55 |
| 3A | SNP | 1125772\|F\|0-45:T>C-45:T>C |  |  |  |  |  |  |  |  |  |  |  |  | 58.4 | 32.2 | 2.92 |
| 3A | SNP | 2275820\|F\|0-7:T>C-7:T>C |  |  |  |  |  |  |  |  |  |  |  |  | -58.4 | 32.2 | 2.92 |
| 3A | SNP | 1673384\|F\|0-8:C>T-8:C>T |  |  |  |  |  |  |  |  |  | -54 | 25.1 | 2.34 |  |  |  |
| 3A | SNP | 1200653\|F\|0-5:G>A-5:G>A |  |  |  |  |  |  |  |  |  |  |  |  | -58.4 | 32.2 | 2.92 |
| 3A | SNP | 1070416\|F\|0-56:G>C-56:G>C |  |  |  |  |  |  |  |  |  |  |  |  | -58.4 | 32.2 | 2.92 |
| 3A | SNP | 5339736\|F\|0-42:C>G-42:C>G |  |  |  |  |  |  |  |  |  |  |  |  | 58.4 | 32.2 | 2.92 |
| 3A | SNP | 1005033\|F\|0-11:G>A-11:G>A |  |  |  |  |  |  | 63 | 23.2 | 2.19 |  |  |  |  |  |  |
| 3A | SNP | 1113363\|F\|0-49:C>T-49:C>T |  |  |  |  |  |  |  |  |  |  |  |  | 58.4 | 32.2 | 2.92 |
| 3A | SNP | 7336185\|F\|0-14:G>A-14:G>A |  |  |  |  |  |  | -66.9 | 24.6 | 2.30 |  |  |  |  |  |  |
| 3A | SNP | 1091277\|F\|0-11:G>C-11:G>C |  |  |  |  |  |  | -62.8 | 22.1 | 2.11 |  |  |  |  |  |  |
| 3A | SNP | 1247288\|F\|0-48:C>T-48:C>T |  |  |  |  |  |  |  |  |  |  |  |  | -57.4 | 31.7 | 2.88 |
| 3A | SNP | 2265573\|F\|0-50:G>A-50:G>A |  |  |  |  |  |  | -62.3 | 22.3 | 2.12 |  |  |  |  |  |  |
| 3A | SNP | 5580210\|F\|0-9:G>A-9:G>A |  |  |  |  |  |  |  |  |  |  |  |  | -61.6 | 36.2 | 3.27 |
| 3A | SNP | 1085403\|F\|0-46:C>T-46:C>T |  |  |  |  |  |  |  |  |  |  |  |  | -58.4 | 32.2 | 2.92 |
| 3A | SNP | 7331406\|F\|0-18:T>G-18:T>G |  |  |  |  |  |  |  |  |  |  |  |  | 58.8 | 32.6 | 2.96 |
| 3A | SNP | 1036835\|F\|0-30:C>A-30:C>A |  |  |  | -43.4 | 22.7 | 2.15 |  |  |  |  |  |  |  |  |  |
| 3A | SNP | 1212042\|F\|0-21:G>A-21:G>A |  |  |  |  |  |  |  |  |  |  |  |  | -58.4 | 32.2 | 2.92 |
| 3A | SNP | 1125497\|F\|0-30:C>T-30:C>T |  |  |  |  |  |  |  |  |  |  |  |  | -62.7 | 29.1 | 2.67 |
| 3A | SNP | 1695196\|F\|0-57:G>C-57:G>C |  |  |  |  |  |  |  |  |  |  |  |  | -58.4 | 32.2 | 2.92 |
| 3A | SNP | 1045911\|F\|0-49:C>T-49:C>T |  |  |  |  |  |  |  |  |  |  |  |  | -50.4 | 23.5 | 2.22 |
| 3A | SNP | 1046207\|F\|0-61:G>T-61:G>T |  |  |  |  |  |  |  |  |  |  |  |  | -58.4 | 32.2 | 2.92 |
| 3A | SNP | 1123319\|F\|0-20:G>C-20:G>C |  |  |  |  |  |  |  |  |  |  |  |  | -65.4 | 32.1 | 2.91 |
| 3A | SNP | 983153\|F\|0-32:T>C-32:T>C |  |  |  |  |  |  |  |  |  |  |  |  | -58.4 | 32.2 | 2.92 |
| 3A | SNP | 1056037\|F\|0-38:A>C-38:A>C |  |  |  |  |  |  |  |  |  |  |  |  | -52.4 | 21.1 | 2.04 |
| 3A | SNP | 999513\|F\|0-50:C>T-50:C>T |  |  |  |  |  |  |  |  |  |  |  |  | -58.4 | 32.2 | 2.92 |
| 3A | SNP | 1021514\|F\|0-34:G>T-34:G>T |  |  |  |  |  |  |  |  |  |  |  |  | 58.4 | 32.2 | 2.92 |
| 3A | SNP | 992268\|F\|0-13:T>C-13:T>C |  |  |  |  |  |  |  |  |  |  |  |  | -58.4 | 32.2 | 2.92 |
| 3A | SNP | 1020174\|F\|0-33:A>G-33:A>G |  |  |  |  |  |  |  |  |  |  |  |  | -58.4 | 32.2 | 2.92 |
| 3A | SNP | 1092266\|F\|0-44:C>T-44:C>T |  |  |  |  |  |  |  |  |  |  |  |  | -58.4 | 32.2 | 2.92 |
| 3A | SNP | 26674207\|F\|0-46:G>T-46:G>T |  |  |  |  |  |  |  |  |  |  |  |  | 49.5 | 22.6 | 2.15 |
| 3A | SNP | 992059\|F\|0-14:G>T-14:G>T |  |  |  |  |  |  |  |  |  |  |  |  | -61.6 | 36.2 | 3.27 |
| 3B | SilicoDArT | 1094473 |  |  |  |  |  |  |  |  |  |  |  |  | -57 | 23.4 | 2.21 |
| 3B | SilicoDArT | 4991836 |  |  |  |  |  |  | 71.1 | 28.3 | 2.60 |  |  |  |  |  |  |
| 3B | SilicoDArT | 1268612 |  |  |  | -42.9 | 21.2 | 2.04 |  |  |  |  |  |  |  |  |  |
| 3B | SilicoDArT | 2308093 |  |  |  |  |  |  |  |  |  | -67.9 | 35.6 | 3.22 |  |  |  |
| 3B | SilicoDArT | 1121012 |  |  |  |  |  |  |  |  |  |  |  |  | -58.4 | 32.2 | 2.92 |
| 3B | SilicoDArT | 1165396 | -40.9 | 34.6 | 3.13 |  |  |  |  |  |  |  |  |  |  |  |  |
| 3B | SilicoDArT | 1087911 |  |  |  |  |  |  |  |  |  |  |  |  | -52.7 | 21.5 | 2.06 |
| 3B | SilicoDArT | 1106554 |  |  |  |  |  |  |  |  |  | 59.1 | 28.6 | 2.62 |  |  |  |
| 3B | SilicoDArT | 1062204 | -40.8 | 30.4 | 2.77 |  |  |  |  |  |  |  |  |  |  |  |  |
| 3B | SilicoDArT | 3022497 |  |  |  |  |  |  | -62.8 | 22.7 | 2.15 |  |  |  |  |  |  |
| 3B | SilicoDArT | 1342165 |  |  |  |  |  |  |  |  |  | -50.7 | 21.6 | 2.07 |  |  |  |
| 3B | SilicoDArT | 1300351 | -37.5 | 22.8 | 2.16 |  |  |  |  |  |  |  |  |  |  |  |  |
| 3B | SilicoDArT | 1108205 |  |  |  |  |  |  | -63.8 | 22 | 2.10 |  |  |  | -63.4 | 37.2 | 3.36 |
| 3B | SilicoDArT | 1055089 |  |  |  |  |  |  | 62.1 | 21.5 | 2.06 |  |  |  |  |  |  |
| 3B | SNP | 3023178\|F\|0-21:A>G-21:A>G |  |  |  |  |  |  |  |  |  |  |  |  | 58.4 | 32.2 | 2.92 |
| 3B | SNP | 26673456\|F\|0-48:A>G-48:A>G |  |  |  |  |  |  |  |  |  |  |  |  | -58.4 | 32.2 | 2.92 |
| 3B | SNP | 1216278\|F\|0-11:A>G-11:A>G |  |  |  |  |  |  |  |  |  |  |  |  | -56.6 | 31.1 | 2.83 |
| 3B | SNP | 1218327\|F\|0-41:G>T-41:G>T |  |  |  |  |  |  |  |  |  |  |  |  | -56.6 | 31.1 | 2.83 |
| 3B | SNP | 1126088\|F\|0-9:C>T-9:C>T |  |  |  | -46.2 | 25.1 | 2.34 | -65.4 | 23.3 | 2.20 |  |  |  | -57.7 | 30.1 | 2.74 |
| 3B | SNP | 1096126\|F\|0-38:A>G-38:A>G |  |  |  |  |  |  |  |  |  |  |  |  | -64.5 | 36.6 | 3.31 |
| 3B | SNP | 1140612\|F\|0-31:G>C-31:G>C |  |  |  |  |  |  |  |  |  |  |  |  | -61.3 | 35.9 | 3.25 |
| 3B | SNP | 1091161\|F\|0-11:C>G-11:C>G |  |  |  |  |  |  |  |  |  |  |  |  | -58.4 | 32.2 | 2.92 |
| 3B | SNP | 1072851\|F\|0-13:A>G-13:A>G |  |  |  |  |  |  |  |  |  |  |  |  | -58.4 | 32.2 | 2.92 |
| 3B | SNP | 1020069\|F\|0-64:G>A-64:G>A |  |  |  |  |  |  | 65.8 | 24.7 | 2.31 |  |  |  |  |  |  |
| 3B | SNP | 7353635\|F\|0-43:T>C-43:T>C | -37.3 | 24.8 | 2.32 |  |  |  |  |  |  |  |  |  |  |  |  |
| 3D | SilicoDArT | 1279746 | -40.8 | 37.1 | 3.35 |  |  |  |  |  |  |  |  |  |  |  |  |
| 3D | SilicoDArT | 1007010 |  |  |  |  |  |  | -62.8 | 22.7 | 2.16 |  |  |  |  |  |  |
| 3D | SilicoDArT | 1218075 |  |  |  |  |  |  | 64 | 23.1 | 2.19 |  |  |  |  |  |  |
| 3D | SilicoDArT | 3532820 |  |  |  |  |  |  |  |  |  |  |  |  | -58.4 | 32.2 | 2.92 |
| 3D | SilicoDArT | 2323639 |  |  |  |  |  |  |  |  |  |  |  |  | -58.4 | 32.2 | 2.92 |
| 3D | SNP | 7155773\|F\|0-13:C>T-13:C>T |  |  |  |  |  |  |  |  |  |  |  |  | -62.7 | 29.1 | 2.67 |
| 3D | SNP | 2246092\|F\|0-39:T>C-39:T>C |  |  |  |  |  |  | -66.9 | 24.6 | 2.30 |  |  |  |  |  |  |
| 3D | SNP | 12772586\|F\|0-64:G>A-64:G>A |  |  |  | -42.3 | 21.3 | 2.05 |  |  |  |  |  |  |  |  |  |
| 3D | SNP | 1011260\|F\|0-51:T>C-51:T>C |  |  |  |  |  |  |  |  |  |  |  |  | -56.1 | 24.9 | 2.32 |
| 3D | SNP | 4009917\|F\|0-7:G>A-7:G>A |  |  |  |  |  |  | 63 | 23.2 | 2.19 |  |  |  |  |  |  |
| 4A | SilicoDArT | 1118611 |  |  |  | -51.9 | 26.3 | 2.43 |  |  |  |  |  |  | -54.5 | 21.1 | 2.03 |
| 4A | SilicoDArT | 977362 |  |  |  |  |  |  |  |  |  |  |  |  | -60.9 | 27.3 | 2.52 |
| 4A | SilicoDArT | 4543602 |  |  |  |  |  |  |  |  |  |  |  |  | -61.8 | 35.1 | 3.17 |
| 4A | SilicoDArT | 1110996 |  |  |  |  |  |  | -72.7 | 31.8 | 2.89 |  |  |  |  |  |  |
| 4A | SilicoDArT | 3941595 |  |  |  | -45 | 20.7 | 2.01 |  |  |  |  |  |  |  |  |  |
| 4A | SilicoDArT | 1090092 | -32.6 | 21.4 | 2.06 |  |  |  |  |  |  |  |  |  |  |  |  |
| 4A | SilicoDArT | 4260828 |  |  |  |  |  |  |  |  |  |  |  |  | -58 | 26.8 | 2.48 |
| 4A | SilicoDArT | 1259415 |  |  |  |  |  |  | 68.6 | 22.9 | 2.17 |  |  |  |  |  |  |
| 4A | SilicoDArT | 1108971 |  |  |  |  |  |  | -72.2 | 23.4 | 2.21 |  |  |  |  |  |  |
| 4A | SilicoDArT | 3534072 |  |  |  |  |  |  |  |  |  |  |  |  | 51.9 | 24.6 | 2.30 |
| 4A | SilicoDArT | 2261220 |  |  |  |  |  |  |  |  |  |  |  |  | -49.8 | 22.9 | 2.17 |
| 4A | SilicoDArT | 1126749 |  |  |  |  |  |  |  |  |  |  |  |  | -48.9 | 21.3 | 2.05 |
| 4A | SilicoDArT | 4009314 |  |  |  | 44.9 | 22.2 | 2.11 |  |  |  |  |  |  |  |  |  |
| 4A | SilicoDArT | 1096623 |  |  |  | -42.7 | 21.8 | 2.09 |  |  |  |  |  |  | -48.2 | 20.7 | 2.00 |
| 4A | SilicoDArT | 1268149 |  |  |  |  |  |  |  |  |  |  |  |  | -52.1 | 20.9 | 2.02 |
| 4A | SilicoDArT | 2261944 |  |  |  |  |  |  |  |  |  |  |  |  | -60.9 | 27.3 | 2.52 |
| 4A | SilicoDArT | 1256664 |  |  |  | -42.2 | 22 | 2.10 |  |  |  |  |  |  |  |  |  |
| 4A | SNP | 1074568\|F\|0-19:G>A-19:G>A |  |  |  |  |  |  |  |  |  |  |  |  | -49.9 | 23 | 2.18 |
| 4A | SNP | 1091678\|F\|0-40:C>A-40:C>A |  |  |  |  |  |  |  |  |  |  |  |  | 56 | 30 | 2.74 |
| 4B | SilicoDArT | 1085634 |  |  |  |  |  |  |  |  |  | -48.8 | 21.7 | 2.08 |  |  |  |
| 4B | SilicoDArT | 1236224 | 38.1 | 26 | 2.41 |  |  |  |  |  |  |  |  |  |  |  |  |
| 4B | SilicoDArT | 1110303 |  |  |  | -56.3 | 37.2 | 3.36 | -71.2 | 26.9 | 2.49 |  |  |  |  |  |  |
| 4B | SilicoDArT | 1237198 |  |  |  |  |  |  |  |  |  | 49.4 | 23.2 | 2.20 |  |  |  |
| 4B | SNP | 1054888\|F\|0-59:T>G-59:T>G |  |  |  |  |  |  |  |  |  | -51 | 23.1 | 2.19 |  |  |  |
| 4B | SNP | 2265458\|F\|0-29:T>C-29:T>C |  |  |  |  |  |  |  |  |  | -68.7 | 36.6 | 3.30 |  |  |  |
| 4D | SilicoDArT | 3954517 |  |  |  |  |  |  |  |  |  |  |  |  | -58.3 | 27.2 | 2.51 |
| 4D | SilicoDArT | 1213810 | -34.2 | 21.7 | 2.08 |  |  |  |  |  |  |  |  |  |  |  |  |
| 4D | SilicoDArT | 1061755 |  |  |  | 48.7 | 29.5 | 2.69 |  |  |  |  |  |  |  |  |  |
| 4D | SilicoDArT | 1165617 |  |  |  |  |  |  |  |  |  | 50.8 | 21.7 | 2.08 |  |  |  |
| 5A | SilicoDArT | 3385219 | 31.8 | 21 | 2.03 |  |  |  |  |  |  |  |  |  |  |  |  |
| 5A | SilicoDArT | 3948527 | 31.8 | 21 | 2.03 |  |  |  |  |  |  |  |  |  |  |  |  |
| 5A | SilicoDArT | 1040969 |  |  |  |  |  |  | -61.6 | 21.9 | 2.10 |  |  |  |  |  |  |
| 5A | SilicoDArT | 1241735 |  |  |  |  |  |  |  |  |  | 48.5 | 22.3 | 2.13 |  |  |  |
| 5A | SilicoDArT | 2303271 |  |  |  |  |  |  |  |  |  | -51.2 | 25.2 | 2.35 |  |  |  |
| 5A | SilicoDArT | 1211121 |  |  |  |  |  |  |  |  |  | 54.7 | 28.3 | 2.60 |  |  |  |
| 5A | SilicoDArT | 7352212 |  |  |  |  |  |  |  |  |  | -52 | 21.2 | 2.04 |  |  |  |
| 5A | SilicoDArT | 7492449 |  |  |  | -43.7 | 20.8 | 2.01 |  |  |  |  |  |  | -67.4 | 40.4 | 3.66 |
| 5A | SilicoDArT | 4543114 |  |  |  |  |  |  |  |  |  | -48.5 | 22.3 | 2.13 |  |  |  |
| 5A | SilicoDArT | 1409358 | -34.4 | 25.4 | 2.36 |  |  |  |  |  |  |  |  |  |  |  |  |
| 5A | SNP | 1133812\|F\|0-10:C>G-10:C>G |  |  |  |  |  |  |  |  |  | -50.7 | 22.8 | 2.17 |  |  |  |
| 5A | SNP | 39570107\|F\|0-36:C>A-36:C>A |  |  |  |  |  |  | -60.1 | 20.7 | 2.00 |  |  |  |  |  |  |
| 5A | SNP | 1089121\|F\|0-29:A>G-29:A>G |  |  |  | -42.1 | 21 | 2.02 |  |  |  |  |  |  |  |  |  |
| 5B | SilicoDArT | 3958080 |  |  |  | -46.7 | 22.6 | 2.15 |  |  |  |  |  |  |  |  |  |
| 5B | SilicoDArT | 1393328 |  |  |  |  |  |  |  |  |  | -54 | 25 | 2.33 |  |  |  |
| 5B | SilicoDArT | 2327548 |  |  |  |  |  |  |  |  |  | -69.2 | 43.7 | 3.98 |  |  |  |
| 5B | SilicoDArT | 49091664 |  |  |  |  |  |  |  |  |  | 54.7 | 21.7 | 2.08 |  |  |  |
| 5B | SilicoDArT | 1181890 | -34 | 24.3 | 2.28 |  |  |  |  |  |  |  |  |  |  |  |  |
| 5B | SilicoDArT | 2276520 | -33.7 | 24.2 | 2.27 |  |  |  |  |  |  |  |  |  |  |  |  |
| 5B | SilicoDArT | 3532692 | 32.5 | 21.2 | 2.04 |  |  |  |  |  |  |  |  |  |  |  |  |
| 5B | SilicoDArT | 1289856 |  |  |  | -46.6 | 24.2 | 2.27 |  |  |  |  |  |  |  |  |  |
| 5B | SilicoDArT | 32940283 |  |  |  |  |  |  |  |  |  | -49.8 | 23.7 | 2.23 |  |  |  |
| 5B | SilicoDArT | 1077993 | -33.2 | 23.4 | 2.21 |  |  |  |  |  |  |  |  |  |  |  |  |
| 5B | SilicoDArT | 3021204 | 36.6 | 28.8 | 2.63 |  |  |  |  |  |  |  |  |  |  |  |  |
| 5B | SNP | 1064158\|F\|0-24:T>C-24:T>C |  |  |  | -42.3 | 22.1 | 2.11 |  |  |  |  |  |  |  |  |  |
| 5B | SNP | 7343312\|F\|0-8:G>A-8:G>A |  |  |  |  |  |  |  |  |  |  |  |  | 51.6 | 25.2 | 2.35 |
| 5D | SilicoDArT | 2275351 |  |  |  |  |  |  |  |  |  |  |  |  | -55.6 | 26.1 | 2.42 |
| 5D | SilicoDArT | 2259640 |  |  |  |  |  |  |  |  |  | -60.5 | 36.9 | 3.33 |  |  |  |
| 5D | SilicoDArT | 3945512 | 36.3 | 23.2 | 2.19 |  |  |  |  |  |  |  |  |  |  |  |  |
| 5D | SNP | 4262574\|F\|0-9:G>A-9:G>A | 32.3 | 21.8 | 2.09 |  |  |  |  |  |  |  |  |  |  |  |  |
| 5D | SNP | 1096556\|F\|0-41:G>C-41:G>C |  |  |  |  |  |  |  |  |  | -48.5 | 22.3 | 2.13 |  |  |  |
| 6A | SilicoDArT | 1113234 |  |  |  | -43.7 | 23.9 | 2.25 |  |  |  |  |  |  |  |  |  |
| 6A | SilicoDArT | 5410397 |  |  |  |  |  |  |  |  |  |  |  |  | -49.6 | 21.2 | 2.04 |
| 6A | SilicoDArT | 3956606 | -38.2 | 23.8 | 2.24 |  |  |  |  |  |  |  |  |  |  |  |  |
| 6A | SilicoDArT | 2300272 |  |  |  |  |  |  |  |  |  |  |  |  | -49.6 | 21.2 | 2.04 |
| 6A | SilicoDArT | 1085072 |  |  |  |  |  |  |  |  |  | -56.5 | 29.3 | 2.68 |  |  |  |
| 6A | SilicoDArT | 1669342 |  |  |  |  |  |  | 92.5 | 41.1 | 3.72 |  |  |  |  |  |  |
| 6A | SilicoDArT | 1266923 | -35.4 | 26.7 | 2.47 |  |  |  |  |  |  |  |  |  |  |  |  |
| 6A | SilicoDArT | 2276633 | -43.4 | 31.9 | 2.90 |  |  |  |  |  |  |  |  |  |  |  |  |
| 6A | SilicoDArT | 2330150 |  |  |  |  |  |  | 65.2 | 23.2 | 2.19 |  |  |  |  |  |  |
| 6A | SilicoDArT | 1006701 |  |  |  | -45.2 | 22.6 | 2.15 |  |  |  |  |  |  |  |  |  |
| 6A | SNP | 12771979\|F\|0-9:G>A-9:G>A | 35 | 23 | 2.18 |  |  |  |  |  |  |  |  |  |  |  |  |
| 6A | SNP | 980448\|F\|0-68:G>C-68:G>C |  |  |  | -46 | 21.8 | 2.09 |  |  |  | -60 | 29.6 | 2.70 |  |  |  |
| 6B | SilicoDArT | 2277596 | 35.7 | 27.5 | 2.53 |  |  |  |  |  |  |  |  |  |  |  |  |
| 6B | SilicoDArT | 1218174 |  |  |  | 49.3 | 23.3 | 2.20 |  |  |  |  |  |  |  |  |  |
| 6B | SilicoDArT | 3935979 |  |  |  | 49.3 | 23.3 | 2.20 |  |  |  |  |  |  |  |  |  |
| 6B | SilicoDArT | 1101618 |  |  |  |  |  |  |  |  |  | -56.3 | 23.3 | 2.20 |  |  |  |
| 6B | SilicoDArT | 1124325 |  |  |  |  |  |  | 67.1 | 23.4 | 2.21 |  |  |  |  |  |  |
| 6B | SilicoDArT | 1228147 |  |  |  |  |  |  |  |  |  | -49.4 | 23.3 | 2.20 |  |  |  |
| 6B | SilicoDArT | 2325221 |  |  |  |  |  |  |  |  |  | -53.7 | 22.9 | 2.17 |  |  |  |
| 6B | SilicoDArT | 4260968 |  |  |  |  |  |  |  |  |  |  |  |  | -55.7 | 27.7 | 2.55 |
| 6B | SilicoDArT | 1221108 |  |  |  |  |  |  | 64.1 | 22.3 | 2.12 |  |  |  |  |  |  |
| 6B | SilicoDArT | 1083469 |  |  |  | -45 | 22.3 | 2.12 |  |  |  |  |  |  |  |  |  |
| 6B | SilicoDArT | 2264223 |  |  |  | 49.7 | 23.7 | 2.24 |  |  |  |  |  |  |  |  |  |
| 6B | SilicoDArT | 1130477 |  |  |  |  |  |  |  |  |  | -51.6 | 20.9 | 2.02 |  |  |  |
| 6B | SilicoDArT | 2260529 |  |  |  |  |  |  | 70.5 | 22.1 | 2.11 |  |  |  |  |  |  |
| 6B | SilicoDArT | 1082408 | 35.3 | 26.9 | 2.48 |  |  |  |  |  |  |  |  |  |  |  |  |
| 6B | SilicoDArT | 4992744 |  |  |  | -55.4 | 30.5 | 2.77 |  |  |  | -62.9 | 30 | 2.74 |  |  |  |
| 6B | SilicoDArT | 3960667 |  |  |  |  |  |  | -76.1 | 26.5 | 2.45 |  |  |  | -63 | 29.5 | 2.70 |
| 6B | SilicoDArT | 5358860 |  |  |  | -51.9 | 35.4 | 3.20 |  |  |  |  |  |  |  |  |  |
| 6B | SilicoDArT | 1073034 | 38.3 | 29.8 | 2.72 |  |  |  |  |  |  |  |  |  |  |  |  |
| 6B | SilicoDArT | 2260226 |  |  |  | -46 | 23.6 | 2.22 |  |  |  |  |  |  |  |  |  |
| 6B | SilicoDArT | 2276003 |  |  |  |  |  |  |  |  |  | -52.2 | 26.5 | 2.45 |  |  |  |
| 6B | SilicoDArT | 1112343 |  |  |  |  |  |  |  |  |  | -49 | 21.1 | 2.03 |  |  |  |
| 6B | SilicoDArT | 3026201 |  |  |  |  |  |  |  |  |  | -56.3 | 23.3 | 2.20 |  |  |  |
| 6B | SilicoDArT | 1093975 |  |  |  |  |  |  | -68.2 | 24.3 | 2.28 |  |  |  |  |  |  |
| 6B | SilicoDArT | 1145710 |  |  |  |  |  |  | -74.2 | 27.4 | 2.53 |  |  |  |  |  |  |
| 6B | SilicoDArT | 2280984 | -38.3 | 29.8 | 2.72 |  |  |  |  |  |  |  |  |  |  |  |  |
| 6B | SilicoDArT | 1025848 | 33.8 | 23.4 | 2.21 |  |  |  |  |  |  |  |  |  |  |  |  |
| 6B | SilicoDArT | 4990659 |  |  |  |  |  |  |  |  |  |  |  |  | 48.8 | 22.1 | 2.11 |
| 6B | SilicoDArT | 1004332 |  |  |  | -49.3 | 23.3 | 2.20 |  |  |  |  |  |  |  |  |  |
| 6B | SilicoDArT | 4408965 |  |  |  | -49.3 | 23.3 | 2.20 |  |  |  |  |  |  |  |  |  |
| 6B | SilicoDArT | 26674177 |  |  |  |  |  |  | -69.3 | 23.5 | 2.22 |  |  |  |  |  |  |
| 6B | SNP | 2279771\|F\|0-36:G>C-36:G>C |  |  |  | -49.3 | 23.3 | 2.20 |  |  |  |  |  |  |  |  |  |
| 6B | SNP | 1069615\|F\|0-24:C>T-24:C>T |  |  |  | -49.3 | 23.3 | 2.20 |  |  |  |  |  |  |  |  |  |
| 6B | SNP | 989458\|F\|0-18:A>G-18:A>G |  |  |  | -49.3 | 23.3 | 2.20 |  |  |  |  |  |  |  |  |  |
| 6B | SNP | 1708133\|F\|0-12:T>A-12:T>A |  |  |  | 49.3 | 23.3 | 2.20 |  |  |  |  |  |  |  |  |  |
| 6B | SNP | 1098568\|F\|0-14:T>C-14:T>C |  |  |  | 43.6 | 20.7 | 2.01 | 69.5 | 25.4 | 2.37 |  |  |  |  |  |  |
| 6B | SNP | 1028370\|F\|0-37:G>A-37:G>A | -32.8 | 22.4 | 2.13 |  |  |  |  |  |  |  |  |  |  |  |  |
| 6D | SilicoDArT | 1118035 |  |  |  |  |  |  |  |  |  |  |  |  | -49.9 | 21.5 | 2.07 |
| 6D | SilicoDArT | 1112370 | -35.8 | 24.2 | 2.27 |  |  |  |  |  |  |  |  |  |  |  |  |
| 6D | SilicoDArT | 1110566 | -38.2 | 23.8 | 2.24 |  |  |  |  |  |  |  |  |  |  |  |  |
| 6D | SilicoDArT | 992969 |  |  |  |  |  |  |  |  |  | -49.9 | 23.8 | 2.24 |  |  |  |
| 6D | SilicoDArT | 1166767 |  |  |  |  |  |  |  |  |  | 61.2 | 36.5 | 3.30 |  |  |  |
| 6D | SilicoDArT | 3960158 |  |  |  |  |  |  |  |  |  | -49.9 | 23.8 | 2.24 |  |  |  |
| 6D | SilicoDArT | 1128748 |  |  |  | -60.8 | 41.1 | 3.73 |  |  |  |  |  |  |  |  |  |
| 6D | SNP | 3020999\|F\|0-11:A>T-11:A>T |  |  |  |  |  |  |  |  |  |  |  |  | -57.1 | 31.4 | 2.85 |
| 6D | SNP | 2264731\|F\|0-39:T>A-39:T>A | -32.8 | 22.4 | 2.13 |  |  |  |  |  |  |  |  |  |  |  |  |
| 6D | SNP | 1018411\|F\|0-65:C>A-65:C>A |  |  |  |  |  |  | 72.2 | 23.5 | 2.21 |  |  |  |  |  |  |
| 6D | SNP | 1091441\|F\|0-17:C>G-17:C>G |  |  |  | -46 | 21.8 | 2.09 |  |  |  | -60 | 29.6 | 2.70 |  |  |  |
| 7A | SilicoDArT | 4260829 | -40.5 | 32.1 | 2.91 |  |  |  |  |  |  |  |  |  |  |  |  |
| 7A | SilicoDArT | 1263225 | -40 | 29 | 2.65 |  |  |  |  |  |  |  |  |  |  |  |  |
| 7A | SilicoDArT | 1240001 |  |  |  |  |  |  |  |  |  | -50.6 | 22.7 | 2.16 |  |  |  |
| 7A | SilicoDArT | 1100092 | -32.9 | 22.5 | 2.14 |  |  |  |  |  |  |  |  |  |  |  |  |
| 7A | SilicoDArT | 1238619 |  |  |  |  |  |  |  |  |  |  |  |  | -49.8 | 21.4 | 2.06 |
| 7A | SilicoDArT | 4439685 |  |  |  |  |  |  |  |  |  | -52.7 | 25 | 2.34 |  |  |  |
| 7A | SilicoDArT | 4992482 |  |  |  |  |  |  | -72.2 | 23.4 | 2.21 |  |  |  |  |  |  |
| 7A | SilicoDArT | 4404669 | -41.1 | 31 | 2.82 |  |  |  |  |  |  |  |  |  |  |  |  |
| 7A | SilicoDArT | 33847371 | 33.3 | 21.7 | 2.08 |  |  |  |  |  |  |  |  |  |  |  |  |
| 7A | SilicoDArT | 5374199 |  |  |  |  |  |  |  |  |  |  |  |  | -61.6 | 36.2 | 3.27 |
| 7A | SilicoDArT | 5411965 |  |  |  |  |  |  |  |  |  | -62 | 29.1 | 2.66 |  |  |  |
| 7A | SilicoDArT | 1667183 |  |  |  |  |  |  | -66.9 | 23.3 | 2.20 |  |  |  |  |  |  |
| 7A | SNP | 1093343\|F\|0-22:G>A-22:G>A |  |  |  |  |  |  |  |  |  | -55.6 | 24.8 | 2.32 |  |  |  |
| 7A | SNP | 1125725\|F\|0-63:G>A-63:G>A |  |  |  |  |  |  |  |  |  |  |  |  | -51 | 24.6 | 2.30 |
| 7B | SilicoDArT | 989589 |  |  |  |  |  |  |  |  |  | 50.2 | 24.2 | 2.27 |  |  |  |
| 7B | SilicoDArT | 987204 |  |  |  |  |  |  |  |  |  | 54.2 | 21.2 | 2.04 |  |  |  |
| 7B | SilicoDArT | 1074102 |  |  |  |  |  |  |  |  |  | 50 | 23 | 2.18 |  |  |  |
| 7B | SilicoDArT | 6024835 |  |  |  |  |  |  |  |  |  |  |  |  | -56 | 29.3 | 2.68 |
| 7B | SilicoDArT | 2291978 | -39 | 25 | 2.33 |  |  |  |  |  |  |  |  |  |  |  |  |
| 7B | SilicoDArT | 1381883 |  |  |  |  |  |  |  |  |  | 60.9 | 27.9 | 2.56 |  |  |  |
| 7B | SilicoDArT | 1109003 |  |  |  |  |  |  |  |  |  | -54.9 | 24.1 | 2.27 |  |  |  |
| 7B | SilicoDArT | 16657372 | -37.1 | 22.2 | 2.12 |  |  |  |  |  |  |  |  |  |  |  |  |
| 7B | SilicoDArT | 1207574 |  |  |  | -42.3 | 21.3 | 2.05 |  |  |  |  |  |  |  |  |  |
| 7B | SilicoDArT | 1261931 |  |  |  | -41.5 | 21.2 | 2.04 |  |  |  |  |  |  |  |  |  |
| 7B | SilicoDArT | 1120154 |  |  |  |  |  |  |  |  |  | 49.8 | 23.7 | 2.23 |  |  |  |
| 7B | SilicoDArT | 5411574 |  |  |  |  |  |  |  |  |  | -60.2 | 36.6 | 3.30 |  |  |  |
| 7B | SilicoDArT | 3935310 |  |  |  |  |  |  |  |  |  |  |  |  | -53.6 | 25.5 | 2.37 |
| 7B | SilicoDArT | 13376136 |  |  |  |  |  |  |  |  |  | -54.2 | 26.6 | 2.46 |  |  |  |
| 7B | SilicoDArT | 1097155 |  |  |  |  |  |  |  |  |  | 49.8 | 23.7 | 2.23 |  |  |  |
| 7B | SNP | 67730130\|F\|0-31:T>C-31:T>C |  |  |  |  |  |  |  |  |  | -58.5 | 34.3 | 3.10 |  |  |  |
| 7B | SNP | 2279895\|F\|0-27:C>G-27:C>G |  |  |  | -44.7 | 24.2 | 2.27 |  |  |  |  |  |  |  |  |  |
| 7B | SNP | 1093097\|F\|0-42:G>C-42:G>C |  |  |  |  |  |  |  |  |  | -51.8 | 25.7 | 2.39 |  |  |  |
| 7B | SNP | 1094727\|F\|0-46:T>C-46:T>C | -32.8 | 20.8 | 2.01 |  |  |  |  |  |  |  |  |  |  |  |  |
| 7B | SNP | 1262636\|F\|0-10:G>T-10:G>T |  |  |  | -42.3 | 22.1 | 2.11 |  |  |  |  |  |  |  |  |  |
| 7B | SNP | 3064589\|F\|0-57:T>C-57:T>C |  |  |  | 47.8 | 21.6 | 2.07 |  |  |  |  |  |  |  |  |  |
| 7B | SNP | 2276097\|F\|0-11:A>G-11:A>G |  |  |  | -44.7 | 24.9 | 2.32 |  |  |  |  |  |  |  |  |  |
| 7D | SilicoDArT | 4543387 | -36.7 | 29.3 | 2.68 |  |  |  |  |  |  |  |  |  |  |  |  |
| 7D | SilicoDArT | 1066402 |  |  |  |  |  |  |  |  |  |  |  |  | -52 | 20.8 | 2.01 |
| 7D | SilicoDArT | 29429446 | -34.3 | 23.3 | 2.20 |  |  |  |  |  |  |  |  |  |  |  |  |
| 7D | SilicoDArT | 1111235 |  |  |  | -43.9 | 21.1 | 2.03 |  |  |  |  |  |  |  |  |  |
| 7D | SilicoDArT | 1102216 |  |  |  |  |  |  |  |  |  |  |  |  | 56.1 | 24.8 | 2.32 |
| 7D | SilicoDArT | 5350250 | 41.1 | 31 | 2.82 |  |  |  |  |  |  |  |  |  |  |  |  |
| 7D | SilicoDArT | 3937740 |  |  |  |  |  |  | -71.8 | 23.1 | 2.19 |  |  |  |  |  |  |
| 7D | SNP | 987784\|F\|0-55:T>G-55:T>G | -36.4 | 23.3 | 2.20 |  |  |  |  |  |  |  |  |  |  |  |  |
| 7D | SNP | 1093097\|F\|0-12:C>G-12:C>G |  |  |  |  |  |  |  |  |  | -51.8 | 25.7 | 2.39 |  |  |  |
| 7D | SNP | 5324104\|F\|0-35:C>G-35:C>G |  |  |  |  |  |  | -67.4 | 21.9 | 2.10 |  |  |  |  |  |  |
